# Delineating functional principles of the bow tie structure of a kinase-phosphatase network in the budding yeast

**DOI:** 10.1186/s12918-017-0418-0

**Published:** 2017-03-16

**Authors:** Diala Abd-Rabbo, Stephen W. Michnick

**Affiliations:** 1 Département de Biochimie et Médecine Moléculaire, Université de Montréal, C.P. 6128, Succursale centre-ville, Montréal, Québec H3C 3J7 Canada; 2 Centre Robert-Cedergren, Bio-Informatique et Génomique, Université de Montréal, C.P. 6128, Succursale centre-ville, Montréal, Québec H3C 3J7 Canada

**Keywords:** Kinase-phosphatase signalling network, Network hierarchical structure, Topological properties, Biological properties, Vertex Sort algorithm, Functional principles of cell behaviour, *Saccharomyces cerevisiae*

## Abstract

**Background:**

Kinases and phosphatases (KP) form complex self-regulating networks essential for cellular signal processing. In spite of having a wealth of data about interactions among KPs and their substrates, we have very limited models of the structures of the directed networks they form and consequently our ability to formulate hypotheses about how their structure determines the flow of information in these networks is restricted.

**Results:**

We assembled and studied the largest bona fide kinase-phosphatase network (KP-Net) known to date for the yeast *Saccharomyces cerevisiae*. Application of the vertex sort (VS) algorithm on the KP-Net allowed us to elucidate its hierarchical structure in which nodes are sorted into top, core and bottom layers, forming a bow tie structure with a strongly connected core layer. Surprisingly, phosphatases tend to sort into the top layer, implying they are less regulated by phosphorylation than kinases. Superposition of the widest range of KP biological properties over the KP-Net hierarchy shows that core layer KPs: (i), receive the largest number of inputs; (ii), form bottlenecks implicated in multiple pathways and in decision-making; (iii), and are among the most regulated KPs both temporally and spatially. Moreover, top layer KPs are more abundant and less noisy than those in the bottom layer. Finally, we showed that the VS algorithm depends on node degrees without biasing the biological results of the sorted network. The VS algorithm is available as an R package (https://cran.r-project.org/web/packages/VertexSort/index.html).

**Conclusions:**

The KP-Net model we propose possesses a bow tie hierarchical structure in which the top layer appears to ensure highest fidelity and the core layer appears to mediate signal integration and cell state-dependent signal interpretation. Our model of the yeast KP-Net provides both functional insight into its organization as we understand today and a framework for future investigation of information processing in yeast and eukaryotes in general.

**Electronic supplementary material:**

The online version of this article (doi:10.1186/s12918-017-0418-0) contains supplementary material, which is available to authorized users.

## Background

To maintain normal homeostasis, living cells continuously accommodate changes to their internal and external environment via signalling pathways. Protein KPs play an essential regulatory role in signalling pathways through phosphorylation and dephosphorylation interactions (PDI) that cause profound effects on substrates, affecting their turnover, localization and interactions with other proteins [1].

Numerous efforts have been made to reconstruct the budding yeast KP-Net from various types of interactions [2–7]. Despite these efforts, KP-Nets assembled so far are not fully mature to represent genuine networks in which a KP acts directly on its substrate for the following reasons. First, dephosphorylation interactions are underrepresented in KP-Nets, because on one hand, dephosphorylation interactions are poorly annotated in public databases (Additional file 1: Table S1) and on the other hand, phosphatases have been modestly studied in comparison to kinases. Second, kinase networks that were assembled from in vitro phosphorylation interactions do not include phosphatases and contain a considerable number of false positives due to non-specific phospohorylation of proteins by kinases in vitro [5–7]. Finally, KP-Nets that were assembled from protein-protein interactions and from genetic interactions, and KP-Nets that were built by knocking out a KP lack two crucial properties: causality and directionality [2–4]. These crucial properties characterize the command-execution aspect of regulatory networks. Causality determines which KP directly acts on which substrate, whereas directionality indicates the direction of the interaction between the two interactors, which is required when substrates are themselves KPs. Interestingly, KP-Nets assembled from high quality PDIs are not characterized by the previously mentioned drawbacks and hence describe better genuine KP-Nets. Despite the large number of KP-Net studies, to our knowledge, no investigations in the budding yeast included in vivo interactions characterized by both causality and directionality [2–4]. KP-Net studies that did include interactions characterized by both causality and directionality were not performed in vivo and did not include phosphatases [5–7] (Additional file 1: Table S2). Hence, constructing a bona fide KP-Net remains an essential goal for analysis of signalling networks.

There have been a number of efforts to determine rules governing the organization and function of biological regulatory networks. For instance, a number of studies invoke command-execution organization characterizing directed networks to elucidate their hierarchical structure using network decomposition methods on various regulatory networks [5, 6, 8–13]. Decomposition methods classify network nodes into different layers to elucidate information flow in network hierarchies. The majority of these efforts were aimed at transcription networks, but rarely at other regulatory networks, including KP networks. In addition, network layers in these studies were characterized by topological and rarely by biological properties of their nodes; that is, KP-Nets are rarely characterized according to the features of the gene products that represent nodes such as stability, abundance and noise in mRNA and protein gene products (Additional file 1: Table S2). However, biological properties are the ones that profoundly affect the regulatory state of any biological network.

Despite the wealth of available evidence, deciphering the complexity of KP-Nets to gain insights into their functional principles is still challenging. Here, we overcame two basic gaps in knowledge in previous studies: first, we constructed the largest bona fide KP-Net for the yeast *Saccharomyces cerevisiae*. Second, we elucidated the KP-Net hierarchical structure using the VS algorithm and unprecedentedly, we integrated the widest range of KP biological properties within this hierarchy in order to describe the functional principles of the KP-Net with our current knowledge. We found that the KP-Net has a bow tie hierarchy formed of three layers (top, core and bottom) and that the different biological properties of KPs are unevenly distributed among KP-Net layers. This uneven distribution reveals general biological properties of KPs in each layer from which we could postulate the behaviours and information processing functions of each layer in the KP-Net hierarchy. We suggest that high protein abundances and low protein noise in KP-Net top layer could result in signal fidelity, whereas enrichment for decision-making and bottleneck proteins in the core layer may underlie signal integration. Finally, we showed that node degrees affect the way the VS algorithm sorts nodes within a network but we also showed that our results and conclusions are not biased by node degrees. We developed an R package called the VertexSort to facilitate VS algorithm application to other networks (https://cran.r-project.org/web/packages/VertexSort/index.html).

## Results

### The kinase-phosphatase network (KP-Net)

**Fig. 1:**
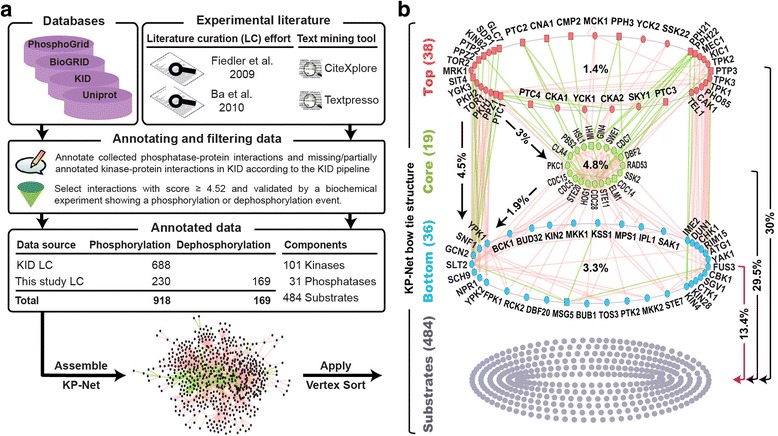
The pipeline used to assemble and to sort the KP-Net, and the KP-Net bow tie structure. **a** The steps followed to elucidate the KP-Net hierarchical structure starting from the different sources used to collect kinase-protein and phosphatase-protein interactions, passing through the data annotation procedure and filtering criteria applied to select high quality PDIs, to the assembly and sorting of the KP-Net by the VS algorithm. **b** The bow tie structure of the KP-Net showing how KPs are classified in top, core and bottom layers. Top layer KPs control core layer KPs; top and core layer KPs control bottom layer KPs and KPs in the three layers control proteins in the substrates layer formed of proteins that are not KPs and of KPs having no substrates. Numbers between parentheses represent number of nodes in each layer. Arrows represent directed interactions (*red*: phosphorylation, *green*: dephosphorylation and *black*: both). Percentages designate percentage of interactions within and between layers

The kinase interaction database (KID) provides the most detailed and specialized annotation of kinase-protein interactions; its annotation is based on 31 experimental categories including genetic, biochemical, physical and phenotypic experimental evidence [14]. However, phosphatase-protein interactions are not included in and many kinase-protein interactions are missing or partially annotated in this database. Hence, we collected these interactions from different sources, then, curated, annotated and scored the collected interactions according to the KID database pipeline with minor adjustments to annotate phosphatase-protein interactions (Fig. 1a and Additional file 1: Supplementary Methods) [2, 15–20]. The KID pipeline associates a confidence score to each interaction based on the extent to which the different experimental methods that validate an interaction contribute to identifying a true positive Kinase-protein interaction. To ensure that the interactions assembled in the KP-Net represent PDIs rather than simply Kinase-protein or phosphatase-protein interactions, we selected interactions having a confidence score ≥ 4.52 (corresponding to a *P* ≤ 5 × 10^−2^) and those validated by at least one biochemical experiment showing the occurrence of a PDI (in vitro kinase assay, in vivo or in vitro phosphosite mapping, mobility shift of phosphoproteins on gel or substrate trapping by a dead phosphatase catalytic domain). The assembled KP-Net contains 1,087 directed interactions (918 and 169 PDIs, respectively) implicating 616 proteins [101 kinases and 31 phosphatases, covering ~77% of these enzymes and 484 proteins, most of which are KP substrates that are not KPs, (Fig. 1a and Additional file 2)]. Similar to other biological networks, the KP-Net possesses a scale-free structure (P(K) ~ K^-2.58^ with a goodness-of-fit test *P* = 1.3 × 10^−2^) in which most KPs regulate few proteins and few KP hubs regulate a large number of proteins (Additional file 1: Supplementary Methods and Figure S1).

### The KP-Net possesses a “corporate” hierarchical structure in the form of a bow tie with a strongly connected core layer

We assessed the amount of the hierarchical structure of the KP-Net by calculating its global reaching centrality (GRC), which represents a normalized average of the proportions of nodes accessible from each node in the network [21]. The closer the GRC is to 1, the more hierarchical the network is. The KP-Net has a moderate GRC of 0.61, suggesting that the KP-Net represents a hierarchical structure that could be placed between two extremes: (i) an autocratic structure comparable to a complete tree and (ii) a democratic structure in which collaborative regulation dominates and no hierarchy exists [5]. Bhardwaj et al. observed a similar moderate hierarchy in a co-phosphorylation network and described it as a corporate hierarchy [5]. Obviously, the KP-Net does not represent a complete tree, as it is enriched for many logic motifs that do not occur in trees: feed-forward loops (a structure in which a node regulates another node and together they regulate a third one), two node feedback loops (two nodes that regulate each other), and bi-fans (a structure in which two nodes regulate two other nodes) (*P* < 10^−3^, Methods). Moreover, the KP-Net does not represent democracies and encapsulates a hierarchical structure, as its GRC is significantly higher than that of Erdős–Rényi random networks (non-hierarchical networks) having the same number of nodes and edges as the KP-Net (*P* < 10^−4^, Methods). Interestingly, the GRC of the KP-Net is significantly smaller than that of random networks generated by degree preserving randomization (DPR, Methods). This result is not surprising, as the degree distribution of a network is essential to determine its organizational structure, meaning networks having same degree distributions will have similar organizational structures. Thus the GRC of the KP-Net was expected to be comparable to that of DPR networks, but it was found to be significantly smaller than the GRC of DPR networks, probably indicating enrichment for feedback loops that generally exist in KP-Nets.

Subsequently, we applied the VS algorithm to the KP-Net to elucidate the network hierarchical structure and the signal flow within the elucidated hierarchy. The VS algorithm is among the best network decomposition algorithms available. It was conceived and applied by Jothi et al. to the transcription regulatory network of the budding yeast *Saccharomyces cerevisiae* to elucidate the network hierarchical structure [5, 6, 8–11, 22]. The VS algorithm sorts nodes into different levels so that nodes in upper levels control those in lower levels [8]. It first transforms a cyclic graph to an acyclic one by collapsing each strongly connected component (SCC, a sub-graph where each node pair is related by two paths of opposite directions) into a super node and then it applies the leaf removal algorithm to the resulting graph and to its transpose. This generates global solutions in which a node could span a range of levels, reflecting the huge amount of missing data in and the dynamic nature of biological networks.

Application of the VS algorithm to the KP-Net revealed a hierarchical structure in which KPs are sorted into 9 levels that we subsequently grouped into three non-overlapping layers: top, core and bottom (Additional file 1: Figure S2a). As in Jothi et al., we first identified KPs of the largest SCC and classified them as belonging to the core layer (19 KPs); we then classified KPs that regulate core layer KPs to the top layer (38 KPs) and those that are regulated by core layer KPs to the bottom layer (36 KPs) (Fig. 1b) [8]. Thirty-eight nodes, of which 33 KPs and five proteins that are not KPs, were excluded from further analysis, because the former are not connected to any KP and the latter are substrates of the excluded KPs (Additional file 1: Figure S2b). The three layers of the KP-Net generated a bow tie structure in which the core layer has relatively fewer nodes than top and bottom layers (Fig. 1b). It is important to note that the bow tie shape of the KP-Net represents an intrinsic property of this network and it is not the result of the application of the VS algorithm. More specifically it is not not the result of choosing the core layer as the SCC of the KP-Net. This is because by applying the VS algorithm in the same way, the hierarchical structure of the regulatory network elucidated by Jothi et al. do not have a bow tie shape (top, core and bottom layers contain 25, 64 and 59 nodes, respectively) [8].

Interestingly, KP-Net top, core and bottom layers regulate 235, 276 and 148 proteins, respectively, corresponding to 38, 45 and 24% of the KP-Net nodes, respectively. Although the core layer is ~2 times smaller in size than top and bottom layers, it regulates a number of substrates that is 1.2 and 1.9 times larger than that regulated by top and bottom layers, respectively, implying an essential role of the core layer in the KP-Net.

### The three layers of the KP-Net have dissimilar biological roles and subcellular localizations

**Fig. 2:**
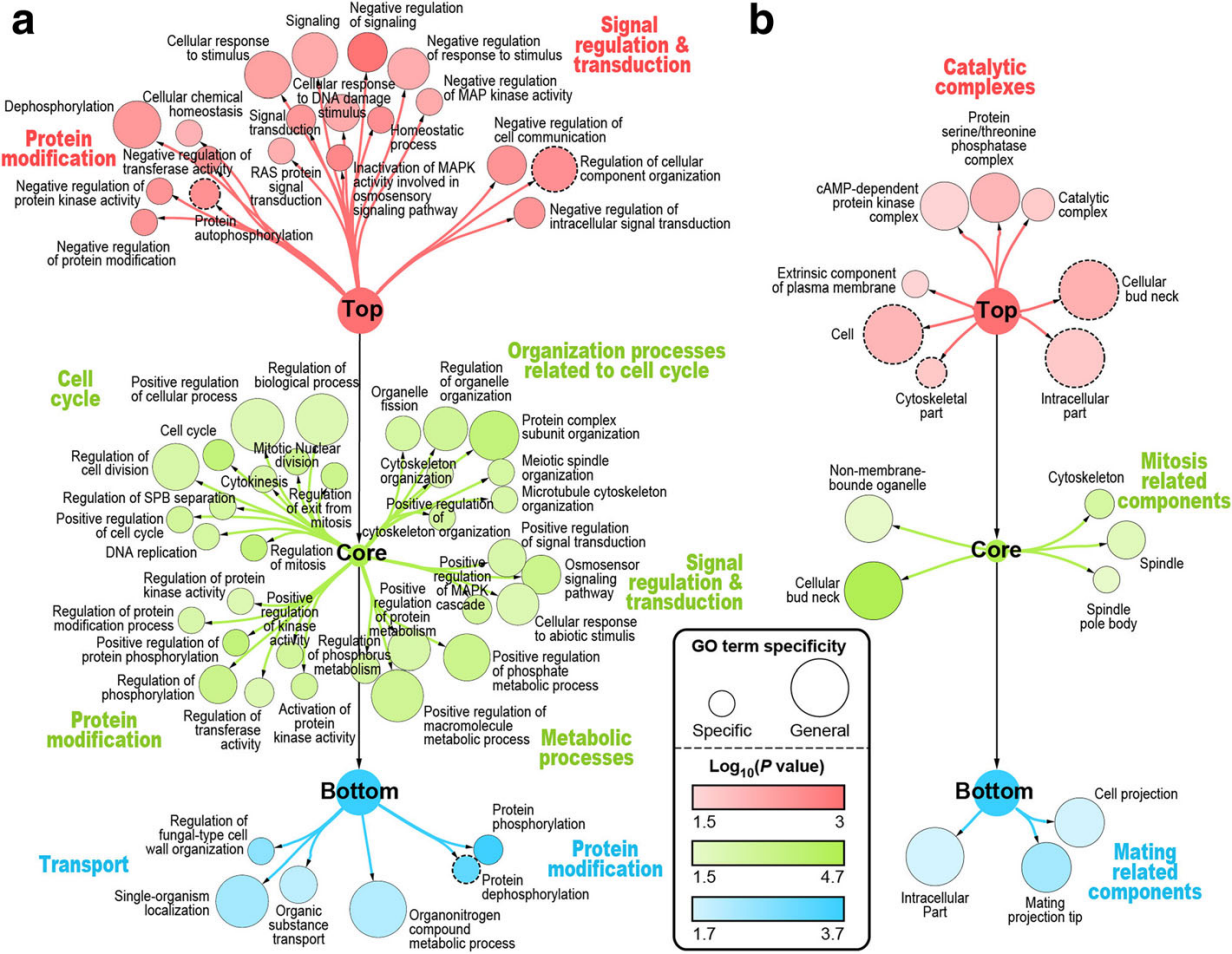
Depleted and enriched biological processes and cellular components in each of the KP-Net layers. Depleted and enriched (**a**) biological processes and (**b**) cellular compartments associated with KPs in each of the KP-Net layers (*top*: *red*; *core*: *green*; and *bottom*: *blue*). Nodes represent the different enriched and depleted GO terms. Colour gradients represent log_10_ of *P*-values (log_10_(*P*)) of enriched and depleted GO terms. Size of nodes indicates the specificity of each GO term (small: specific and large: general). Enriched GO terms are encircled with solid border, while depleted ones are encircled with a dashed border

To unravel biological roles of the KP-Net layers, we performed a Gene Ontology (GO) enrichment/depletion analysis for KPs in each of these layers (Additional file 1: Supplementary Methods). We found that the KP-Net top layer is enriched mostly for signal regulation and transduction; interestingly, the core layer is enriched for signalling also, for metabolic processes, but mostly for cell cycle, organization processes related to cell cycle and decision-making (Additional file 1: Table S3), confirming the essential role of the core layer in the KP-Net; and the bottom layer is enriched for few GO terms, suggesting that it has a less specialized and more diverse biological roles (Fig. 2a). These results are in line with the findings of Bhardwaj et al. [5].

On another level, the top layer is depleted for, whereas the core layer is enriched for KPs located in the bud neck (Fig. 2b), a result that has been already observed by Cheng et al. [6]. We further found that the bottom layer is enriched for KPs located in the mating projection tip (Fig. 2b). The latter observations suggest that top layer KPs might remain in the mother cell to regulate signalling, while core layer KPs may be polarized towards the daughter cell to contribute to mitosis, and bottom layer KPs might reside in the cell projection to contribute in mating.

Strikingly, dephosphorylation is enriched in the top layer and depleted in the bottom layer of the KP-Net (Fig. 2a), suggesting that phosphatases are over-represented in signalling pathway upstream and depleted in downstream arms of signalling pathways. The latter results are consistent with dynamic phosphoproteomic studies showing that at least 50% of early responses to cell perturbations are dephosphorylation of phosphosites [23].

### Phosphatases are less regulated by phosphorylation than kinases

Our findings confirmed our proposition that the top layer is enriched whereas the bottom layer is depleted for phosphatases (Additional file 1: Figure S3a, *P* = 2.2 × 10^−5^ and *P* = 4.1 × 10^−4^ respectively; hypergeometric test (HT)). In addition, we observed that 81% of the top layer phosphatases have a zero in-degree. Using high quality phosphoproteomic data annotated in the PhosphoGRID database, we also found that the number of phosphosites identified in phosphatase protein sequences is smaller than that identified in kinases (Additional file 1: Figure S3b, *P* = 2.3 × 10^−3^; randomization test (RT), Methods). These results suggest that phosphatases are less regulated by phosphorylation than kinases are. Our suggestion is also supported by the great variety of regulatory subunits controlling phosphatases [24] and by the large number of cellular mechanisms, other than phosphorylation, reported to regulate phosphatases, including phosphorylation of the regulatory subunits of phosphatases [25–30].

### KP-Net upper levels are the least regulated and KP-Net lower levels are the least to regulate other KPs

**Fig. 3:**
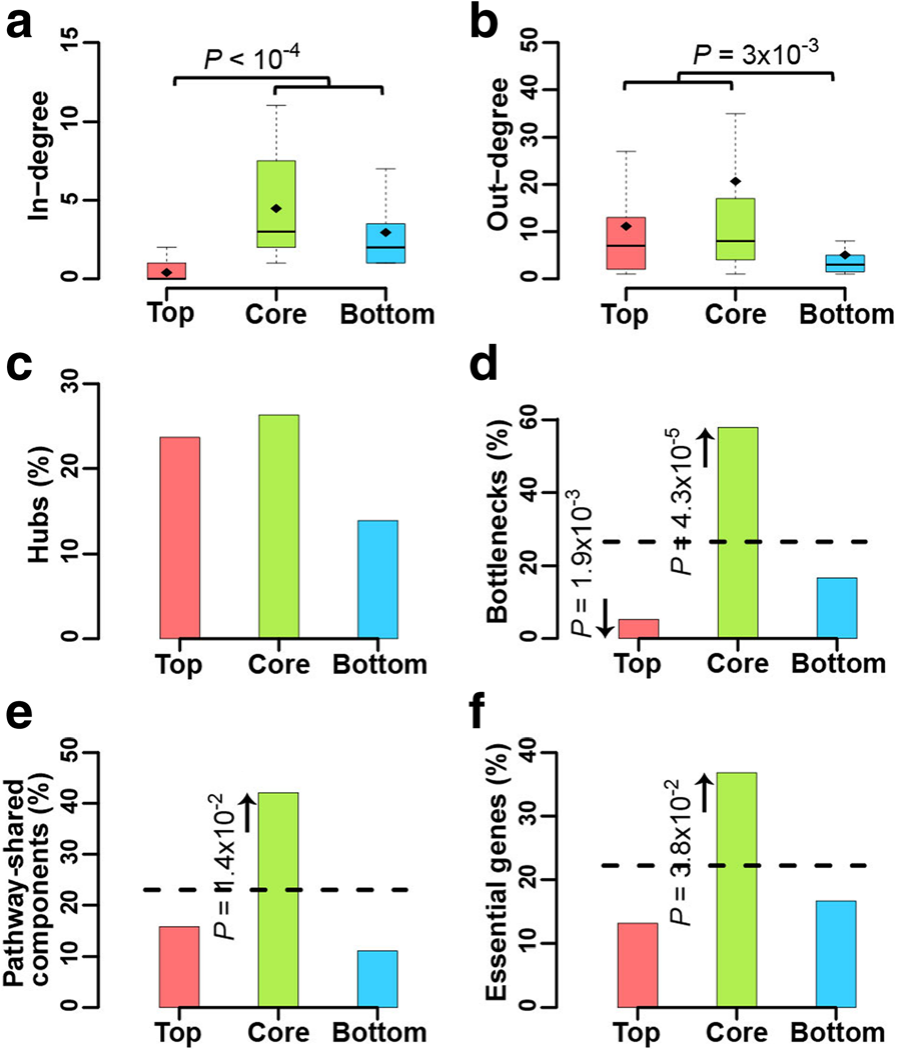
Topological and biological properties of KPs in the different layers of the KP-Net. Distribution of (**a**) in-degree and (**b**) out-degree of KPs in each layer of the KP-Net and percentage of KPs representing (**c**) hubs, (**d**) bottlenecks, (**e**) shared components between pathways (KPs involved in at least two pathways), and (**f**) essential genes in each layer of the KP-Net. The *broken line* in bar plots represents the expected mean of the corresponding percentage in each layer. *Black diamonds* in box plots designate the average of the corresponding property of KPs. Outliers were omitted from box plots to simplify data representation. *P*-values were calculated by comparing property means of two layers and the enrichment/depletion of a property within a layer using the RT (Methods) and HT, respectively. For description of the used datasets see Supplementary Materials in Additional file 1

Top layer KP in-degrees are on average smaller than KP in-degrees in core and bottom layers (Fig. 3a, *P* < 10^−4^; RT, Methods). This observation is a direct result of the VS algorithm application (*P =* 10^−3^; degree non-preserving randomization (DNPR), Methods) to a network, but it agrees with organizational principles found in hierarchical systems in which members of upper levels are the least regulated (e.g. pyramid networks). In contrast, the out-degree of the bottom layer is significantly smaller than that of top and core layers (Fig. 3b, *P =* 3 × 10^−3^; RT, Methods). This finding is independent of the VS algorithm application (*P =* 0.7; DNPR, Methods) on a network and has been previously observed in the hierarchical structure of a yeast transcriptional regulatory network elucidated by a decomposition algorithm (Breadth-First Search) different than the VS algorithm [9]. Finally, the observed features related to node in- and out-degrees were implemented in two network decomposition algorithms, other than the VS algorithm, to classify nodes in top and bottom layers, respectively [5, 12].

### The KP-Net core layer is enriched for essential genes, bottlenecks, and pathway-shared components

To better grasp our knowledge of signal flow in the KP-Net, we analysed the distribution of hubs, bottlenecks, pathway-shared components (KPs involved in at least two pathways) and essential genes in the three layers of the KP-Net. Hubs and bottlenecks are defined as the 20% of KPs in the KP-Net that have, respectively, the highest degree and the highest betweenness (fraction of shortest paths between all pairs of nodes that pass through a single node; this measure captures how much signalling passes through a node). The hubs are equally distributed among the three layers, reflecting the prevalence of parallel regulation as a principle emerging from the three layers of the KP-Net (Fig. 3c). Interestingly, the core layer is enriched for bottlenecks, pathway-shared components and essential genes (Fig. 3d–f, *P* = 4.3 × 10^−5^, *P* = 1.4 × 10^−2^ and *P* = 3.8 × 10^−2^, respectively; HT), suggesting that most of the signal integration and crosstalk between pathways occur in the core layer.

### Molecular switches are enriched in KPs in core and bottom layers

**Fig. 4:**
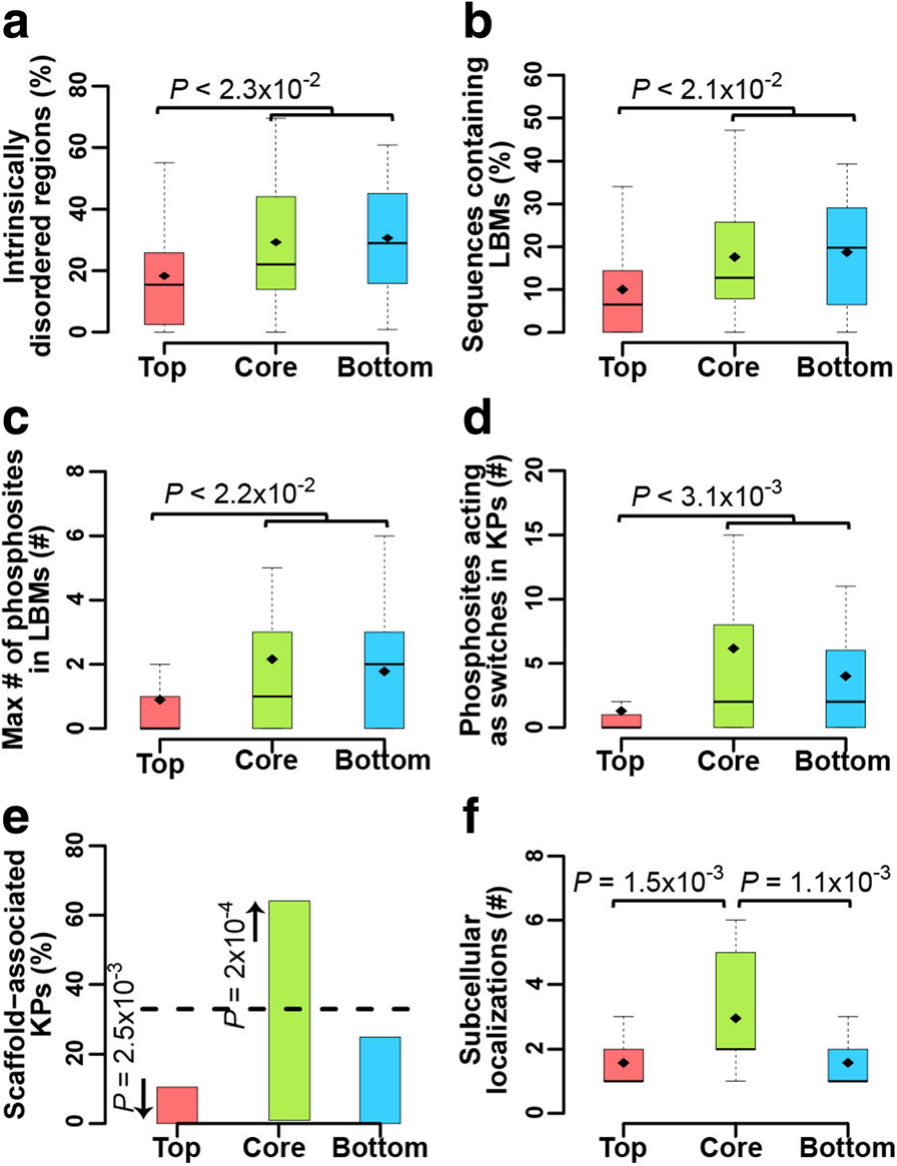
Biochemical and spatiotemporal modulators of KPs in the different layers of the KP-Net. Distribution of (**a**) the percentage of disordered regions, (**b**) the percentage of predicted linear binding motifs (LBM), (**c**) the maximum number of phosphosites within a predicted LBM, (**d**) the number of phosphosites acting as molecular switches in each KP in KP-Net layers and (**e**) the percentage of scaffold-associated KPs and (**f**) the distribution of the number of subcellular localizations in which a KP was detected for KPs in each of the three KP-Net layers. For description of box plots and bar plots, see Fig. 3 and for description of used datasets see Supplementary Materials in Additional file 1

Molecular switches represent phosphosites within or adjacent to linear binding motifs (LBM) which mediate “on demand” controls switching proteins between different functional states (on-off, specificity, cumulative and sequential switches) [31]. Given their fundamental role in controlling signalling networks, we investigated the distribution of KP molecular switches in the KP-Net hierarchy. We predicted protein disordered regions in KP protein sequences and LBMs within these predicted disordered regions using the IUPred and ANCHOR algorithms, respectively (Additional file 1: Supplementary Methods) [32, 33]. We then overlaid bona fide in vivo phosphosites from the PhosphoGRID database on top of KP protein sequences (Additional file 1: Supplementary Materials). We found that percentage of predicted disordered regions in KP proteins in core and bottom layers are on average higher compared to the top layer (Fig. 4a, *P* < 2.3 × 10^−2^; RT, Methods). The same trend is observed for: (i), the percentage of sequences predicted to contain LBMs (Fig. 4b, *P* < 2.1 × 10^−2^; RT, Methods); (ii), the number of phosphosites in KP sequences generally (Additional file 1: Figure S3c, *P* < 6.1 × 10^−4^; RT, Methods) and (iii), in the predicted LBMs particularly (Fig. 4c, *P* < 2.2 × 10^−2^; RT, Methods); and (iv), the number of potential molecular switches in each KP (Fig. 4d, *P* < 3.1 × 10^−3^; RT, Methods). Interestingly, our findings suggest that phosphorylation of KPs in lower layers could form molecular switches important for KP temporal regulation. Two out of many examples confirming our suggestions are: (1) the specificity switch in Hsl1 (core layer kinase and morphogenesis checkpoint regulator) leading to a G2 arrest essential for cell survival upon osmotic shock and (2) the on-switch in Swe1 (core layer kinase) maintaining Cdc28 in an inhibited form essential for entry of cells into mitosis [34, 35].

### Core layer KPs employ scaffolding to prevent unwanted pathway crosstalk

It is well established that redirecting information flow within signalling networks is accomplished through interactions of KP with scaffold proteins and is required for the insulation of interconnected pathways [36]. Interestingly, the KP-Net core layer is enriched for pathway-shared components (Fig. 3e) and for LBMs (Fig. 4b), suggesting that core layer KPs that are shared between pathways associate with scaffold proteins through LBMs. Indeed, although core and bottom layers are enriched for potential LBMs, only the core layer is enriched for scaffold-associated KPs (Fig. 4e, *P* = 2 × 10^−4^; HT). This indicates that scaffolding is extensively employed at the core layer where most pathway crosstalk occurs (Fig. 3d–e), in order to prevent inappropriate cellular responses resulting from the activation of undesired pathways. For instance, the mitogen extracellular signal-regulated kinase kinase Ste11, a core layer kinase, is involved in three pathways: high osmolarity, filamentous growth and pheromone pathway. Association of Pbs2 (a MAPK kinase and a scaffold protein implicated in the HOG signalling pathway) and Ste5 (a pheromone-responsive MAPK scaffold protein) with Ste11 reorients signal flow by activating the HOG signalling pathway and the mating pathway, respectively; whereas, unavailability of both Pbs2 and Ste5 favours filamentous growth [37].

### Core layer KPs undergo more spatial organization changes than top and bottom layer KPs

Controlling spatial distribution of KPs plays an essential role in tuning KP activity and specificity towards their substrates [38, 39]. By superposing microscopic subcellular localization data of proteins in single cells under different stress conditions [40] on top of the KP-Net hierarchy, we observed that KPs in the core layer dynamically redistribute among more subcellular compartments than KPs in top and bottom layers (Fig. 4f, *P* < 1.6 × 10^−3^; RT, Methods). This indicates that core layer KPs might be subject to a more stringent control than top and bottom layer KPs to tightly restrict their localization. Hog1 is a relevant example of a core layer kinase that is translocated from the cytoplasm to the nucleus to trigger a wide transcriptional response on exposure to a high osmolarity stimulus [41]. Another typical example of tight localization control is Cdc14, a core layer phosphatase essential for mitotic exit, which after its sequestration in the nucleolus, is released to the nucleus and the cytoplasm where it associates with the spindle pole body during early anaphase [42].

### Top layer KP proteins are more abundant and less noisy than bottom layer KPs of the KP-Net

**Fig. 5:**
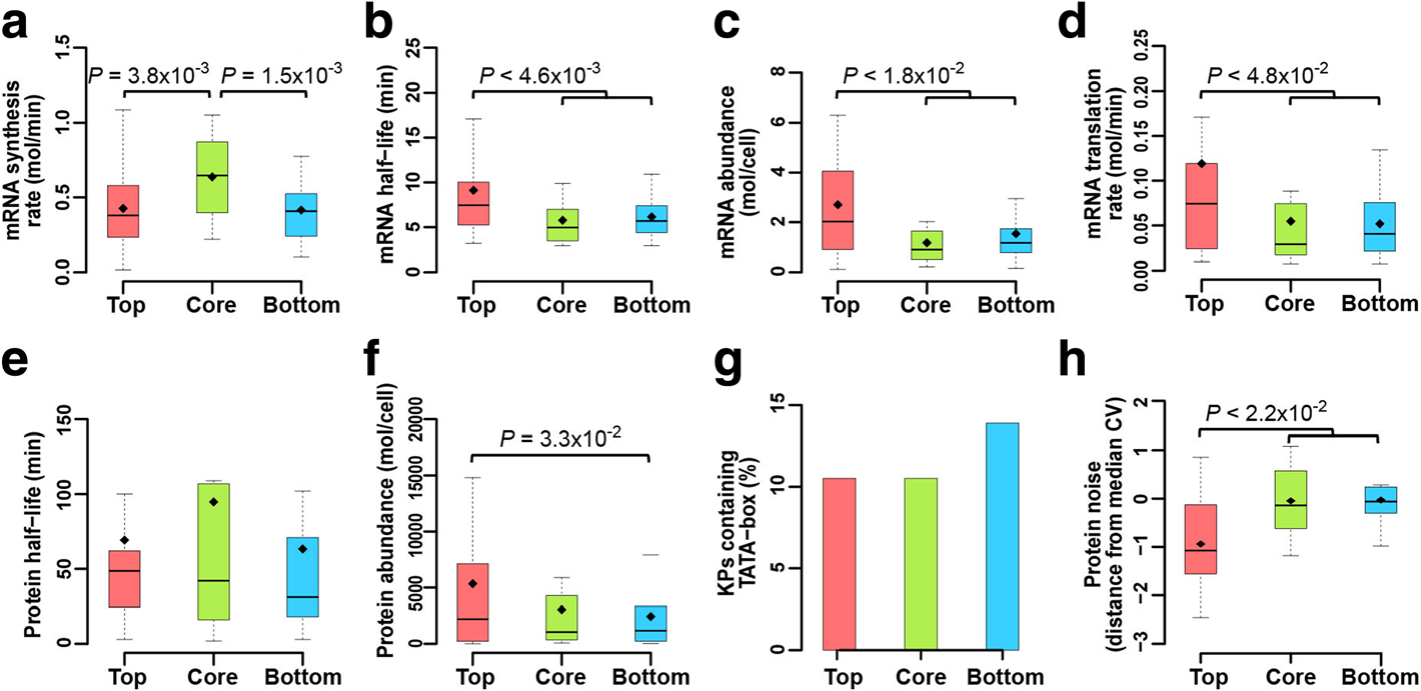
mRNA and protein turnover related properties of KPs in the different layers of the KP-Net. Distribution of (**a**) mRNA synthesis rate, (**b**) mRNA half-life, (**c**) mRNA abundance, (**d**) mRNA translation rate, (**e**) protein half-life, (**f**) protein abundance, (**g**) percentage of noisy mRNA KPs and (**h**) distribution of noise in KP protein abundance of KPs in the different layers of the KP-Net. A KP is considered to be noisy at the transcriptomic level, if the promoter region of its gene was predicted to contain a TATA-box consensus sequence. Protein noise was defined as the distance of coefficient of variation (CV) of protein abundance from a running median of protein abundance CV. For description of box plots and bar plots, see Fig. 3 and for description of used datasets see Supplementary Materials in Additional file 1

Since KPs turnover determines their availability and thus their activity, we overlaid various information of KP turnover taken from the literature (Additional file 1: Supplementary Materials) on top of the KP-Net hierarchy [43–49]. While transcripts coding for core layer KPs are synthesized at a higher rate than top and bottom layers (Fig. 5a, *P* < 3.9 × 10^−3^; RT, Methods), mRNA of top layer KPs have longer half-lives than core and bottom layers (Fig. 5b, *P* < 4.6 × 10^−3^; RT, Methods). However, mRNA abundance has a similar trend to mRNA half-life, implying that mRNA degradation (the process that determines half-lives) is more important than synthesis rate in determining mRNA abundance (Fig. 5c, *P* < 1.8 × 10^−2^; RT, Methods). Similarly, mRNA of top layer KPs are translated at higher rates than core and bottom layers (Fig. 5d, *P* < 4.8 × 10^−2^; RT, Methods). However, half-lives of KP proteins are statistically comparable among the three layers of the KP-Net (Fig. 5e; RT, Methods), suggesting that proteins abundance should have the same trend as the translation rate of mRNA molecules. This is partially true, since top layer KP proteins are more abundant than the bottom layer (Fig. 5f, *P* = 3.3 × 10^−2^; RT, Methods), but not more abundant than the core layer. This discrepancy might be due to the fact that KP proteins in the core layer tend to have longer half-lives (mean values are reported; 95 min, Fig. 5e) than the top layer (69 min, Fig. 5e). On another level, percentages of noisy KP genes at the mRNA level are comparable among the three KP-Net layers (Fig. 5g; HT). Moreover, top layer KP proteins are less noisy than core and bottom layers in starving *S. cerevisiae* cells (Fig. 5h, *P* < 2.2 × 10^−2^; RT, Methods). Interestingly although, we observed significant relative differences in each of protein abundance and noise between KP-Net layers, notably proteins were abundant (Fig. 5f, top 5,336 molecules/cell, core 3,041 molecules/cell and bottom 2,436 molecules/cell) and not noisy (Fig. 5h, top -0.94 a.u., core -0.05 a.u. and bottom 0.12 a.u.) in the three layers of the KP-Net. Taken together, these results suggest that higher protein abundance coupled with lower protein noise in the three layers and in particular in the top layer, might confer high signalling fidelity to the KP-Net.

### The VS algorithm depends on node degree to classify network nodes in three layers

**Fig. 6:**
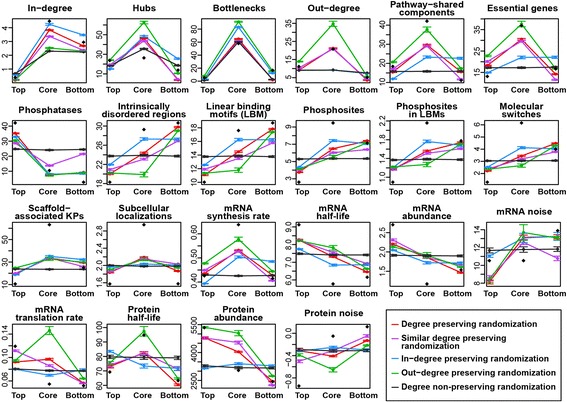
The VS algorithm depends on node degrees to sort network nodes into three layers. The mean and its 95% confidence interval of the studied properties of KPs in the three layers of each of the five sets of the 1,000 random networks generated by: degree preserving randomization (DPR, *red line*), similar degree preserving randomization (SDPR, *pink line*), in-degrees preserving randomization (IDRP, *blue line*), out-degree preserving randomization (ODRP, green line) and degree non-preserving randomization (DNPR, *black line*). The black diamonds represent the mean of studied properties of KPs in the three layers of the KP-Net

As the findings of this study mainly result from the application of the VS algorithm, we asked whether the VS algorithm depends on a specific node property to sort nodes into three layers and whether these findings reflect the biology underlying the KP-Net. To address these questions, we generated five sets of 1,000 random networks produced using five randomization methods: degree preserving randomization (DPR), similar degree preserving randomization (SDPR), in-degree preserving randomization (IDPR), out-degree preserving randomization (ODPR), and degree non-preserving randomization (DNPR) (Methods). We then applied the VS algorithm on these random networks and plotted means of KP properties in each layer of the KP-Net (black diamonds, Fig. 6), means of KP properties in each layer of random networks (points joined by coloured lines, Fig. 6) and the 95% confidence interval of random network means (coloured vertical segments, Fig. 6).

Strikingly, we observed that the distribution of all properties, except in-degrees, hubs and bottlenecks, of the three layers form a straight horizontal line for DNPR networks (Fig. 6, black line), showing that the VS algorithm produces a particular global signature (they peak at the core layer) in completely random networks for only these three properties that are all related to node degrees. Interestingly, the distribution of all properties in the DPR and SDPR networks (red and pink lines, Fig. 6) are the closest to each other when node degrees are similar to each other (DPR and SDPR cluster together in Additional file 1: Figure S4). Taken together, our observations suggest that the VS algorithm depends on node degree to sort network nodes in the different layers. Moreover, on clustering the five sets of randomized networks using the Euclidean distance between the different properties of their KPs, we found that ODPR networks are closer to DPR networks than IDPR networks (Additional file 1: Figure S4), suggesting that the VS algorithm depends on node out-degrees more than node in-degrees. However, the VS algorithm obviously depends also on node in-degrees, as any node with a zero in-degree will be automatically placed in the top layer. Therefore, the VS algorithm depends on both nodes in- and out-degrees. Nevertheless, although the VS algorithm depends on node degrees to classify network nodes into different layers, three observations suggest that KP biological properties are not associated with KP degrees and that they are not the result of a bias in the VS algorithm: (i) all biological properties showed a straight line distribution in completely random networks (Fig. 6, black line); (ii), most of the means of KP biological properties in KP-Net layers (black diamonds, Fig. 6) are outside of the 95% confidence interval of the means of the corresponding properties in random network layers; and (iii), most of the KP biological properties (12 out of 18) are neither associated with their in- nor with their out-degrees (Additional file 1: Supplementary methods).

### Robustness of results and incompleteness of data

**Fig. 7:**
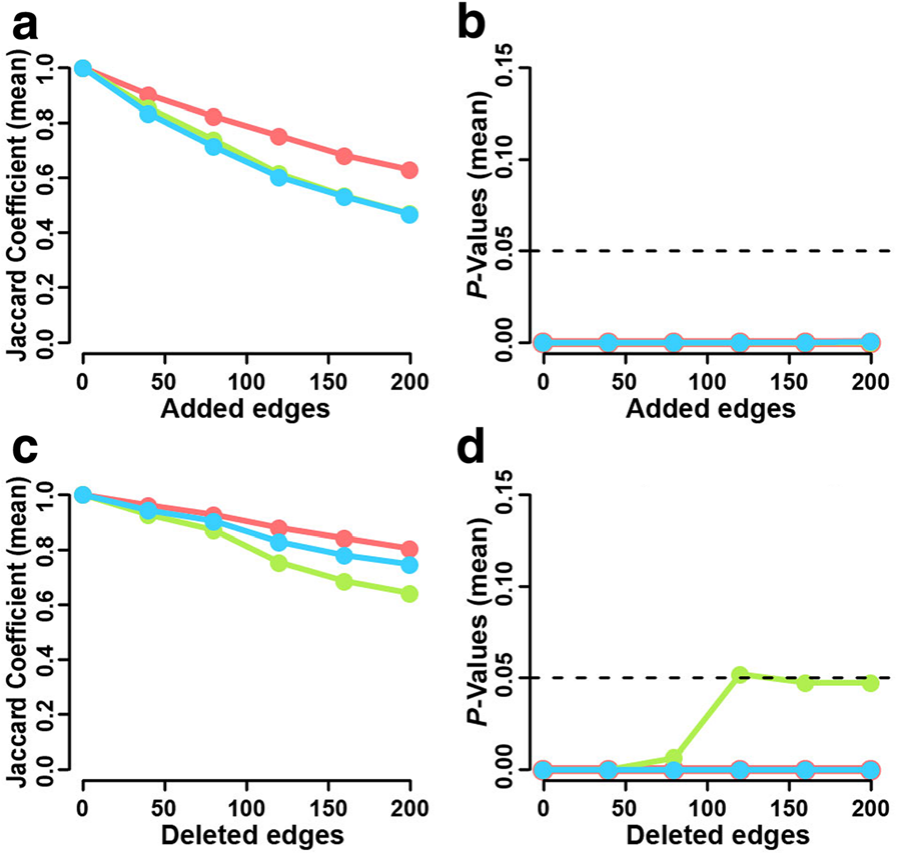
Stability of KP-Net layers and their overlap with subsampled/noisy network layers. **a** Stability of KP-Net layers on adding edges to the KP-Net. **b** Significance of the overlap between KPs in each layer of the KP-Net and noisy network layers on adding edges. **c** Stability of KP-Net layers on deleting edges from the KP-Net. **d** Significance of the overlap between KPs in each layer of the KP-Net and subsampled network layers on deleting edges. Stability was quantified using the Jaccard coefficient as a similarity measure between KPs belonging to KP-Net layers and those belonging to noisy/subsampled network layers. *P*-values in (b) and (d) were calculated using the HT. Colours designate the different layers of the noisy/subsampled networks (*top*: *red*, *core*: *green* and *bottom*: *blue*)

It did not escape our attention that the KP-Net that was assembled in this study represents a small snapshot of the whole phosphorylation network of the budding yeast. Therefore, we assessed the robustness of our results to missing interactions by generating noisy networks (adding edges to the KP-Net) and the robustness of our results to false positives by generating subsampled networks (deleting edges from the KP-Net) (Methods). We then assessed the stability of KP-Net layers using the Jaccard coefficient as a measure of similarity between KP-Net layers and noisy/subsampled network layers (Methods) [50]. Also, we assessed the significance of the overlap between KP-Net layers and noisy/subsampled network layers using the HT (Methods) [50]. We observed that the KP-Net is more robust to removing than to adding edges (Fig. 7a and c). Moreover, the more edges are added to and removed from the KP-Net, the more the three layers become unstable (Fig. 7a and c). However, in spite of this instability, all layers in noisy/subsampled networks significantly overlap with the KP-Net layers (Fig. 7b and d), showing that our findings are sufficiently robust to describe the KP-Net with our current knowledge. Finally, properties characterizing the KP-Net were retained to different degrees in the noisy networks (Additional file 1: Supplementary Methods and Figure S9), confirming that the characteristics of the KP-Net elucidated in this study represent the best of our knowledge to date about KP-Nets.

### Using the KP-Net as a gold standard to predict kinases acting on substrates in the HOG pathway

Presently, one of the most active areas of research consists of linking each KP to its substrates. As an example, we attempted to predict the kinases that could phosphorylate substrates characterized by a change in their level of phosphorylation in cells exposed to osmotic shock. We used the KP-Net as a gold standard; we overlaid on top of it phosphorylation consensus motifs curated from the literature and proteins that undergo time-dependent phosphorylation or dephosphorylation following osmotic shock from Kanshin & Bergeron-Sandoval et al. [23]. We identified 57 interactions linking 19 kinases to 25 potential substrates (Methods and Additional file 3). The overlap between the predicted kinases in our study and the kinases that underwent changes in phosphorylation in Kanshin & Bergeron-Sandoval et al. was significant (*P* = 3.8 × 10^−2^; HT). This result suggests, first, that a significant number of the 19 kinases that we predicted to act on 25 potential substrates do undergo time-dependent changes in phosphorylation that may reflect their activation or deactivation in response to osmotic shock; second, that the interactions forming the KP-Net that was assembled in this study are of high confidence; and finally, that this same KP-Net could be used as a benchmark with other phosphoproteomic data to identify kinases and perhaps phosphatases that act on a set of substrates.

## Discussion

In this study, we assembled the largest bona fide KP-Net known to date for the yeast *Saccharomyces cerevisiae*. We found, first, that the KP-Net has a moderate hierarchical structure made of three layers (top, core and bottom) in the form of a bow tie structure having a strongly connected core layer. Second, phosphatases are for the first time shown to be less directly regulated by kinases than are kinases by each other. Third, the observed high abundance and low noise of KP proteins in the three layers of the KP-Net, but notably in the top layer, may reflect an adaptation by which maximal sensitivity to signals at the earliest steps of signalling is assured. Finally, the tight temporal and spatial regulation that we observed for the core layer of the KP-Net could be explained by both the high load of signals received by this layer and its enrichment for KPs implicated in cell cycle and decision-making.

Recently, Cheng et al. overlaid many of the biological properties studied here on top of a kinase network assembled from in vitro phosphorylation interactions in the budding yeast (Additional file 1: Table S2) [7]. In contrast to our findings, most of the examined biological properties by Cheng et al. were statistically comparable among the three layers (gene essentiality, abundance, half-life and noise on mRNA and protein levels). It is important to note that properties of each layer depend on the identity and the properties of the proteins belonging to each layer. Difference between findings of Cheng et al. and those of this study might be due to the following reasons: (i), the lack of phosphatases in the network analysed by Cheng et al.; (ii), the high number of false positives that normally exist in any data generated in vitro, which could affect sorting of nodes in the different layers and thus directly affect layer properties; (iii), the application of a decomposition method differing from the VS algorithm, (iv), or a combination of all these reasons. Interestingly though, protein noise results of Cheng et al. concord partially with our findings as proteins in the top layer were less noisy than those in the bottom layer.

A limitation of the KP-Net generated in this study is that it cannot be used to predict novel PDIs or pathways. Note, however, that this was not among the objectives of this study. The KP-Net can serve as a gold standard in future investigations of signalling networks to suggest a set of KP candidates that might act on substrates under a given condition, as we showed in predicting kinases that act on substrates following osmotic stress. Another limitation is that although the choice of the largest SCC to represent the core layer was subjective and inspired by previous application of the VS algorithm to a transcription regulatory network, we can justify the validity of our choice by the concordance of our observations with those in the literature [8]. In the literature, a core layer of a bow tie structure is usually associated with critical decisions determining the system outputs [51]. This concords with our findings showing that 79% of the core layer KPs are implicated in cell cycle and decision-making processes, to note that the VS algorithm does not necessarily generate a bow tie structure as in reference [8] (Figure 2a; Additional file 1: Table S3). Finally, the assembled KP-Net represents a small snapshot of the real-world KP-Net affecting 60% of the proteome. Advances in high throughput technologies should eventually complete the KP-Net by unravelling missing PDIs. As with any network reconstruction exercise, there is the risk that a different sorting of KPs within the KP-Net hierarchical structure could lead to different interpretations of the KP-Net. However, when we randomly added edges to the KP-Net in order to create “noisy networks”, we observed that the layers of the noisy KP-Nets became less stable by adding more edges; but at the same time, they overlap significantly with KP-Net layers (Fig. 7a and b). These results show that the properties of the KP-Net layers are robust to describe how the KP-Net functions with the best of our current knowledge, which represents the principal objective of this study.

Despite the limitations mentioned above, the functional principles of the KP-Net that are proposed in this study are consistent with other observations. Interestingly, bow tie structures are frequently associated with robustness against removal of some of their components and to external perturbations [51–54]. Robustness of the KP-Net bow tie structure could be ensured by the following factors. First, the degeneracy (overlapping functions) of many KPs in the top layer [e.g. PKAs, Tel1-Mec1 and calcineurins, (Fig. 1b)] guaranties that failure of a KP to activate a given pathway is buffered by another KP having partially redundant functions [51]. Notably, the degeneracy observed in top layer KPs concords well with the low number (13%) of KPs encoded by essential genes belonging to this layer. Second, the core layer possesses the required features for generating coordinated responses: (i), it receives and integrates various inputs (high node in-degrees and enrichment for bottlenecks, pathway-shared components, and scaffold-associated KPs (Figs. 3a, d, e and 4e); (ii), it occupies a central position in the hierarchy (Fig. 1b); (iii), it is involved in critical tasks (cell cycle and decision-making) (Fig. 2a and Additional file 1: Table S3); and most importantly, (iv) it is highly regulated at different levels in time and space. Without such a tightly regulated layer, coordinated responses would necessitate ample individual controls and any misregulation of the latter controls would easily impair cellular survival [53]. All these characteristics contribute in delineating functional principles of the KP-Net as known to date.

## Conclusions

In this study, we built a KP-Net assembled from high quality PDIs in the budding yeast, determined its hierarchical structure and integrated the widest range of KP biological properties with elucidated hierarchical structure. This allowed us to formulate hypotheses about the functions of the KP-Net layers. As mentioned previously, the KP-Net assembled in this study represents a snapshot of the KP-Net that exists in the budding yeast. Advances in large-scale screens, in particular those exploring substrates of KPs will enhance coverage of the assembled KP-Net. Also, with the enhancement of high throughput technologies, integration of other type of biological properties, such as methylation, ubiquitination, and temporal PDIs, with the KP-Net might become possible, which could reveal new functional principles of the KP-Net. A better perception of how the KP-Net functions could also open new opportunities to understand the actions of KP inhibitors on normal and pathological processes such as cancers.

## Methods

### Over-representation of various logic motifs in the KP-Net

One thousand random networks were generated by degree preserving randomization (DPR, Methods). Each of the random networks was sorted by the VS algorithm and the number of its feed-forward loops, feedback loops, and bi-fan logic motifs was assessed. The *P*-value is the fraction of times the number of each logic motif in random networks is as large as that in the KP-Net.

### Network randomization

In this study, we randomized the KP-Net using five types of network randomizations:

- **Degree preserving randomization (DPR):** we randomly selected two edges of the KP-Net and exchanged their ends. We then removed multiple edges having the same direction between two nodes by switching each of them with randomly selected edges. The rewiring procedure was repeated 10,000 times to each random network.
- **Similar degree preserving randomization (SDPR):** we used the matching algorithm (Methods) to generate random graphs having similar degree distributions to that of the KP-Net [55–57]. We then switched network edges using the first randomization method (DPR) to make sure that the generated random networks differ from each other.
- **In-degree preserving randomization (IDPR):** interactions were represented as a table made of two columns: “from” and “to”. We recreated the “from” column by randomly selecting KPs with replacement. We then switched network edges using the first randomization method (DPR).
- **Out-degree preserving randomization (ODPR):** we recreated the “to” column by randomly selecting KPs with replacement. We then switched network edges using the first randomization method (DPR).
- **Degree non-preserving randomization (DNPR):** we created a random network from scratch by connecting two nodes that were randomly selected with replacement.

### The matching algorithm

In order to generate networks having a degree distribution that is similar to that of the KP-Net, we defined the degree distribution of the random network by randomly selecting three groups of KPs: the first group had the same in- and out-degrees as the KP-Net and the second and third groups had the same in- and out-degrees as the KP-Net, but incremented and decremented by 1, respectively. Second, we connected the network using a variant of the matching algorithm [58]. Briefly, each vertex of the random network was assigned a number of in- and out-stubs equal to its in- and out-degrees. In- and out-stubs were selected in pairs and joined up to make the network edges. In each step, the selection of in- and out-stubs was weighted by the square of the current in- and out-stubs that were not yet connected but should be. This procedure produced random networks that have very similar in- and out-degrees distributions to those of the KP-Net.

### Testing whether the KP-Net GRC is bigger than Erdős–Rényi network GRCs

We generated 10,000 Erdős–Rényi random networks having the same number of nodes and edges as the KP-Net and calculated their GRCs [21]. The *P*-value of this test is the proportion of random network GRCs that are as large as the KP-Net GRC.

### Comparing means of node properties in two layers using RT

Let L1 and L2 be the size of two layers of the KP-Net to be compared; S the set containing the nodes of these layers; S1 the set of L1 nodes randomly sampled without replacement from S; S2 the set of the remaining L2 nodes in S after sampling. The difference between the means of the node properties in S1 and S2 were calculated. These steps were repeated 10,000 times. The *P-value* is equal to the proportion of times the difference between the means of the sampled sets are as big/small as the difference between the means of the two compared layers.

### Generating subsampled/noisy networks and assessing their layers stability and their overlap with KP-Net layers

We generated ten sets of 100 subsampled and noisy networks from the KP-Net. The five sets of subsampled/noisy networks were produced by randomly removing/adding 40, 80, 120, 160 and 200 edges to the KP-Net, respectively. These steps were repeated 100 times, so that each set contains 100 subsampled/noisy networks. We then applied the VS algorithm to each subsampled/noisy network to identify their three layers. Layer stability of the generated networks was assessed using the Jaccard coefficient as a similarity “cluster wise” measure between the original and the subsampled/noisy layers [50]. Overlap between original and subsampled/noisy layers were assessed using the hypergeometric test (HT).

### Predicting kinases

First, we identified substrates in the KP-Net that contain a phosphorylated residue modulated by time after osmotic shock defined as dynamic phosphosite by Kanshin and Bergeron-Sandoval et al. [23]. We also identified the consensus phosphorylation motifs of each kinase in the KP-Net when possible from the literature (Additional file 4). We then connected each substrate containing a dynamic phosphosite to all kinases having a consensus motif matching the substrate phosphosite by edges to form kinase-substrate interactions. Using the KP-Net as a gold standard network, we retained kinase-substrate interactions that occur in the KP-Net.

## Additional files

Additional file 1: Supplementary materials, supplementary methods, supplementary figures and supplementary tables. (DOCX 3242 kb)

Additional file 2: KP-Net phosphorylation and dephosphorylation interactions: Phosphorylation and dephosphorylation interactions (PDI) that were included in the KP-Net and the experimental methods that validated them with the Pubmed references of the articles in which PDIs were reported. The KID database pipeline was used to score and annotate these interactions (Additional file 1: Supplementary Methods). (XLSX 280 kb)

Additional file 3: Predicted kinases implicated in the HOG pathway: The kinase-substrate interactions that were predicted to be implicated in osmotic shock in this study. (XLSX 14 kb)

Additional file 4: Kinase consensus phosphorylation sites: The consensus phosphorylation sites of kinases and their evidence in the literature. (XLSX 16 kb)

## Abbreviations

CV: Coefficient of variation
DNPR: Degree non-preserving randomization
DPR: Degree preserving randomization
GO: Gene ontology
GRC: Global reaching centrality
HT: Hypergeometric test
IDPR: In-degree preserving randomization
KID: Kinase interaction database
KP: Kinase and phosphatase
KP-Net: Kinase-phosphatase network
LBM: Linear binding motif
ODPR: Out-degree preserving randomization
PDI: Phosphorylation and dephosphorylation interaction
RT: Randomization test
SCC: Strongly connected component
SDPR: Similar degree preserving randomization
VS: Vertex sort
