# Supplementary material for: Delineating functional principles of the bow tie structure of a kinase-phosphatase network in the budding yeast
Source: BMC Syst Biol. 2017 Mar 16;11:38. doi: 10.1186/s12918-017-0418-0 (PMC5353956; doi:10.1186/s12918-017-0418-0)
Supplement: Supplementary file 1 — Supplementary materials, supplementary methods, supplementary figures and supplementary tables. (DOCX 3242 kb) [file 12918_2017_418_MOESM1_ESM.docx]

**Additional file 1**

**Delineating Functional Principles of the Bow Tie Structure of a Kinase-Phosphatase Network in the Budding Yeast**

Diala Abd-Rabbo^1,2^ and Stephen Michnick^1,2^*

^1^ Département de Biochimie et Médecine Moléculaire, Université de Montréal, Montréal, Québec, H3C 3J7, Canada.

^2^ Centre Robert-Cedergren, Bio-Informatique et Génomique, Université de Montréal, Montréal, Québec, H3C 3J7, Canada.

* To whom correspondence should be addressed. Tel: +1 (514) 343-5849; Fax: +1 (514) 343-2210; Email: stephen.michnick@umontreal.ca

Table of Materials

[1 Supplementary Materials 2](#_Toc476316030)

[1.1 Gene essentiality 2](#_Toc476316031)

[1.2 Pathway-shared components 3](#_Toc476316032)

[1.3 mRNA synthesis and degradation rates 3](#_Toc476316033)

[1.4 mRNA and protein abundance 3](#_Toc476316034)

[1.5 mRNA translation rates 3](#_Toc476316035)

[1.6 Protein half-lives 3](#_Toc476316036)

[1.7 mRNA and protein noise 3](#_Toc476316037)

[1.8 Protein sequence of KPs 4](#_Toc476316038)

[1.9 Phosphorylation sites in KPs 4](#_Toc476316039)

[1.10 Scaffolds 4](#_Toc476316040)

[2 Supplementary methods 4](#_Toc476316041)

[2.1 Bioinformatics analysis 4](#_Toc476316042)

[2.2 The pipeline used to assemble the KP-Net 4](#_Toc476316043)

[2.3 Inference of the KP-Net degree distribution and finding the function that best fitted the inferred distribution 5](#_Toc476316044)

[2.4 Testing whether random networks have a bow tie structure on sorting them by the VS algorithm 5](#_Toc476316045)

[2.5 Assessing enriched/depleted gene ontology (GO) terms associated with KPs in each layer of the KP-Net 5](#_Toc476316046)

[2.6 Features of in- and out-degrees of nodes in top and bottom layers in random networks sorted by the VS algorithm 6](#_Toc476316047)

[2.7 Predicting disordered regions in KPs 6](#_Toc476316048)

[2.8 Predicting linear binding motifs in disordered regions in KPs 6](#_Toc476316049)

[2.9 Biological properties of KPs are independent of their in- and out-degrees 6](#_Toc476316050)

[2.10 Investigating whether properties characterizing the KP-Net layers are retained in noisy networks 7](#_Toc476316051)

[3 Supplementary Figures 8](#_Toc476316052)

[Figure S1. Distributions of the cumulative degree and of the clustering coefficient of KP-Net nodes follow a power law distribution. 8](#_Toc476316053)

[Figure S2. (a) The detailed hierarchical structure of the KP-Net. 9](#_Toc476316054)

[Figure S3. Percentage of phosphatases in each of the three layers of the KP-Net and distribution of phosphosites among KPs and among the different layers of the KP-Net. 10](#_Toc476316055)

[Figure S4. Clustering of the different sets of randomized networks. 11](#_Toc476316056)

[Figure S5. Correlation between KP in-degrees and KP numerical properties. 12](#_Toc476316057)

[Figure S6. In-degree distribution of KPs characterized and not characterized by each of the studied categorical properties. 13](#_Toc476316058)

[Figure S7. Correlation between KP out-degrees and KP numerical properties. 14](#_Toc476316059)

[Figure S8. Out-degree distribution of KPs characterized and not characterized by each of the studied categorical properties. 15](#_Toc476316060)

[Figure S9. Distribution of the different properties of KPs in each layer of 100 noisy networks. 16](#_Toc476316061)

[4 Supplementary Tables 17](#_Toc476316062)

[Table S1. Annotation of dephosphorylation interactions. 17](#_Toc476316063)

[Table S2. K-Nets and KP-Nets studies. 18](#_Toc476316064)

[Table S3. Core layer KPs implicated in decision-making. 19](#_Toc476316065)

[5 References 20](#_Toc476316066)

# Supplementary Materials

## Gene essentiality

The list of essential genes was downloaded from the Saccharomyces gene database (SGD) as of Aug. 2015 [[1](#_ENREF_1)]. A gene was considered to be essential if it has an inviable phenotype on its deletion, that is the ‘phenotype’ and ‘mutant_type’ columns in the downloaded table are equal to ‘inviable’ and ‘null’, respectively.

## Pathway-shared components

A pathway-shared component is a kinase or phosphatase (KP) in the kinase-phosphatase network (KP-Net) that participates in more than one pathway. Pathways where accessed from the Bioconductor KEGGREST package and queried using the R scripting language [[2-4](#_ENREF_2)].

## mRNA synthesis and degradation rates

mRNA synthesis and degradation rates were obtained from a large scale study that used metabolic labelling to quantify total and newly transcribed mRNAs at 41 time points in *Saccharomyces cerevisiae* cells that were synchronized at G1 phase using the alpha factor [[5](#_ENREF_5)]. Absolute mRNA synthesis and degradation rates were estimated by this study using the comparative dynamic transcriptome analysis (cDTA) [[6](#_ENREF_6)].

## mRNA and protein abundance

mRNA expression profiles were taken from a high-throughput study using a “generalized” adaptor-tagged competitive PCR (GATC-PCR) between genomic DNA and complementary DNA [[7](#_ENREF_7)]. Protein abundances were obtained from the PaxDB database in particle per million (ppm) unit [[8](#_ENREF_8)]. To convert these abundances into mol/cell, we fit a linear regression model between protein abundance from Ghaemmaghami (mol/cel) and integrated abundance from PaxDB (ppm). This resulted in the following equation:

*log_10_(ppm_abund) = log_10_(mol_cel_abund) - 1.9858.*

To get protein abundances in mol/cel unit, we multiplied the ppm integrated abundances by 96.78.

## mRNA translation rates

mRNA translation rates were taken from a genome scale study that provides a profile of ribosome association with mRNAs in rapidly growing budding yeast cells. mRNAs were separated using velocity sedimentation on a sucrose gradient and 14 fractions across this gradient were collected and quantified using DNA microarrays [[9](#_ENREF_9)].

## Protein half-lives

Protein half-lives were obtained from a large scale study that used western blot analysis to assess the time that a TAP-tagged protein takes to be degraded after blocking protein synthesis by cycloheximide [[10](#_ENREF_10)].

## mRNA and protein noise

mRNA noise was obtained from a previous study assessing the probability that a messenger contains a TATA-box consensus sequence [[11](#_ENREF_11)]. The protein noise was taken from the Newman et al. study [[12](#_ENREF_12)] in which the authors measured noise as the coefficient of variation (CV) of protein abundance (the ratio of the protein standard deviation to its mean abundance) in a population of *Saccharomyces cerevisiae* cells growing under a stress condition (in a minimal (SD) media). For every protein, DM represents the difference between the noise value of that protein and a running median of proteins noise. Lower DM values represent less noise in protein abundance.

## Protein sequence of KPs

Protein sequence of KPs were retrieved from the SGD database as of Nov 2014 and analysed using the seqinR R library [[13](#_ENREF_13), [14](#_ENREF_14)].

## Phosphorylation sites in KPs

Phosphorylation sites in KPs were downloaded from the PhosphoGRID database as of Dec. 2014 [[15](#_ENREF_15)]. Only phosphorylation sites that were validated by at least two independent studies were retained.

## Scaffolds

Scaffolds were annotated from the SGD database [[1](#_ENREF_1)].

# Supplementary methods

## Bioinformatics analysis

All analysis were performed using in-house written scripts using the R project for statistical computing environment unless it was indicated otherwise [[3](#_ENREF_3)].

## The pipeline used to assemble the KP-Net

Approximately 75% of the kinase-protein interactions of the KP-Net were collected from the kinase interaction database (KID) [[16](#_ENREF_16)], a kinase specialized database that annotates and scores kinase-protein interactions validated by a broad number of low and large scale experimental methods. Despite the high quality of the KID data, many kinase-protein interactions were reported in literature and missing or not fully annotated in the KID database. Thus, we assembled these interactions as well as phosphatase-protein interactions from BioGRID, PhosphoGrid, Uniprot, two literature curation efforts and using the Textpresso and CiteXplore text mining tools [[17](#_ENREF_17), [15](#_ENREF_15), [18-22](#_ENREF_18)]. We detected the articles reporting these interactions. We then identified the experimental methods used in the articles to validate these interactions and we retrieved the text passages that provide evidence for the interactions of interest. Annotation of each interaction was based on the 31 experimental categories that were used in the KID database. These experimental categories include genetic, physical and phenotypic evidence. We annotated each interaction by including the PubmedID of the article in which the interaction was validated, the first author name of the article, the year of the article and the text passage extracted from the article providing evidence of the interactions. We then scored the collected Kinase-protein and phosphatase-protein interactions according to the KID database annotation pipeline [[16](#_ENREF_16)]. Briefly, we used the log-likelihood ratios that were calculated by Sharifpoor et al. to summarize the weight of each experimental method in identifying a true positive kinase-protein interaction [[16](#_ENREF_16)]. Subsequently, we sum up all these weights to produce a KID score. Finally, we adapted the experimental methods to take into account the dephosphorylation interactions representing the reverse of phosphorylation interactions. For instance, we replaced the experimental method “*in vivo* reduction in phospho-peptide detected by mass spectrometry on mutating the kinase” by “*in vivo* increase in phospho-peptide detected by mass spectrometry on mutating the phosphatase”. Moreover, we added the experimental method “trapping phospho-substrate by dead phosphatase catalytic domain” to the KID validation experimental methods and we associated to it the lowest score (1.2) associated to experimental methods considered as biochemical experiment showing a phosphorylation interaction. These biochemical experiments are marked with a star on top of the column headers in Additional File 2. Then, in order to collect bona fide direct phosphorylation and dephosphorylation interactions (PDI), we selected interactions having a confidence score ≥ 4.52 (corresponding to a *P* ≤ 5 x 10^-2^) and validated by at least one high- or low-throughput biochemical experiment showing the occurrence of a PDI event.

## Inference of the KP-Net degree distribution and finding the function that best fitted the inferred distribution

We tested whether the degree distribution of the KP-Net nodes follows a power law distribution by using the procedure suggested in [[23](#_ENREF_23)]. Briefly, the scaling parameter (alpha) and the lower bound on the scaling region (x_min_) of the power law distribution were estimated using the maximum likelihood method. Then, a goodness-of-fit test was performed between a large number (5,000) of datasets generated by the theoretical power law distribution defined by the estimated parameters and the observed data using the Kolmogorov-Smirnoff test. The *P*-value of the goodness-of-fit test represents the percentage of number of times where the Kolmogorov-Smirnoff test rejected the suggestion that the power law distribution is a plausible hypothesis for the observed data. The same pipeline was used to test whether the empirical data follows an exponential or a normal distribution. The analyses were done using the R code developed by [[24](#_ENREF_24)]. Our results show that the empirical data fits (i) a power law distribution defined by an alpha = 2.58 and x_min_ = 9 with a goodness-of-fit test *P* = 1.3 x 10^-2^, (ii) an exponential distribution defined by a lambda = 0.05 with a goodness-of-fit test *P* = 7.8 x 10^-1^ and (iii) a normal distribution defined by a mean of 2.3 and standard deviation of 0.5 with a goodness-of-fit test *P* = 1. In summary, the degree distribution of the KP-Net best fits a power law distribution or in other words is said scale free (Figure S1).

## Testing whether random networks have a bow tie structure on sorting them by the VS algorithm

We applied the VS algorithm on 1,000 random networks generated from the KP-Net by DPR and identified the three layers in each random network. We then tested whether each of these networks possesses a bow tie structure or in other words whether its core layer has fewer nodes than its top and bottom layers. The *P*-value of this test is the fraction of random networks in which the number of nodes in the core layer is smaller than that of top and bottom layers.

## Assessing enriched/depleted gene ontology (GO) terms associated with KPs in each layer of the KP-Net

This analysis was performed using the conditional hypergeometric test implemented in the Bioconductor GOStats library of the R project for statistical computing environment [[25](#_ENREF_25), [4](#_ENREF_4), [3](#_ENREF_3)]. it consist of testing whether the number of KPs associated to a given GO term is larger/lower than expected by chance in a particular layer compared to the number of KPs in the KP-Net. The conditional hypergeometric test is characterized by taking into consideration the hierarchical structure of the GO terms tree which causes ancestors to inherit their children GO terms, hence limiting the list of over- and under-represented GO terms to the most specific ones.

## Features of in- and out-degrees of nodes in top and bottom layers in random networks sorted by the VS algorithm

We applied the VS algorithm on 1,000 random networks generated from the KP-Net by DNPR and identified the three layers in each random network. We then tested the occurrence of two features: (i), whether the in-degree of nodes in the top layer is smaller than that of nodes in core and bottom layers and (ii), whether the out-degree of nodes in the bottom layer is smaller than that of nodes in top and core layers. The *P-*value of each test is equal to the fraction of random networks having the corresponding tested feature. The first test generated a *P* of 10^-3^, showing that the VS algorithm classifies nodes having the smallest in-degrees in the top layer, and the second test generated a *P* of 0.7, showing that the VS algorithm does not necessarily classify nodes having the smallest out-degrees in the bottom layer.

## Predicting disordered regions in KPs

We used the IUPred algorithm with the long mode option to predict disordered regions in KPs [[26](#_ENREF_26)]. This prediction is based on amino acid interactions energy, as amino acids in disordered regions cannot establish sufficient stabilizing interactions. Each amino acid of KP sequences was associated an IUPred score, indicating whether it occurs in disordered regions or not. Scores greater than or equal to 0.5 are associated to amino acids predicted to be in disordered regions. Disordered regions were defined as sequences composed of amino acids having an IUPred score above 0.5 as used in [[26](#_ENREF_26)].

## Predicting linear binding motifs in disordered regions in KPs

We used the ANCHOR algorithm to predict linear binding motifs in disordered regions in KPs [[27](#_ENREF_27)]. This prediction is also based on amino acid interactions energy, such that amino acids in binding motifs residing in disordered regions are not only unable to establish sufficient stabilizing intra-chain interactions, but are also able to gain stabilizing energy on interacting with a globular domain of a protein partner. Each amino acid of KP sequences was associated an ANCHOR score. Scores above 0.5 are associated to amino acids predicted to be in binding motifs in disordered regions. Putative binding motifs were defined as sequences composed of at least 6 consecutive amino acids having an ANCHOR score above 0.5 as used in [[27](#_ENREF_27)].

## Biological properties of KPs are independent of their in- and out-degrees

To confirm that the observed biological properties of the KP-Net are not the result of a bias in the VS algorithm, we investigated the association between the different studied properties of KPs and their in- and out-degrees, respectively. Unsurprisingly, KP topological properties (hubs and bottlenecks) are associated with their in- and out-degrees, but most of KP biological properties are not or are weakly associated with their in- and out-degrees (Figures S5-S8). Only, the number of KP phosphosites is moderately correlated with KP in-degrees (Figure S5, R = 0.45; Spearman correlation), and the association of KP with scaffold and the type of enzyme (KP) are associated with KP in-degrees (Figure S6, *P* = 2.6 x 10^-3^ and *P* = 3.4 x 10^-2^, respectively; one-way ANOVA test). To note that, the first result is a consequence of the KP-Net definition: the more KP sequences contain phosphosites, the more KPs are phosphorylated or dephosphorylated, thus the higher their in-degree is and vice-versa. The third result was expected, as phosphatases are over-represented in the top layer and they contain less phosphosites than kinases (Figures S3a and S3b), suggesting that phosphatase in-degrees are smaller than kinase in-degrees. Moreover, KP protein abundance and noise are also mildly correlated with their out-degrees (Figure S7, R = 0.35; Spearman correlation) and KP gene essentiality is associated to KP out-degrees (Figure S8, *P* = 1.4 x 10^-3^; one-way ANOVA test). In conclusion, 6 out of 18 KP biological properties are associated with either their in- or out-degrees and most of the KP biological properties (12 out of 18) are neither associated with their in- nor with their out-degrees. Thus, most KP biological properties describing each layer of the KP-Net are intrinsic to the network and are classified independently of KP degrees.

## Investigating whether properties characterizing the KP-Net layers are retained in noisy networks

Since KP-Net interactions are of high confidence, we limited our analysis to investigate whether the properties characterizing the KP-Net layers were retained in noisy networks. On adding 200 edges (~18% of the KP-Net edges) to the KP-Net, most of the topological (in-degree, out-degree and bottlenecks) and 6 out of 18 biological properties (i.e. phosphosites, molecular switches, scaffold-associated KPs, mRNA synthesis rate, mRNA noise and protein degradation) were retained in the noisy networks (Figures S9a, S9b, S9d, S9j, S9l, S9m, S9o, S9r and S9t), 1 out of 4 topological properties and 11 out of 18 biological properties were mildly retained (i.e. hubs, pathway-shared components, phosphatases, intrinsically disordered regions, LBMs, number of phosphosites in LBMs, cellular localizations, mRNA abundance, mRNA translation rate, protein abundance and protein noise, Figures S9c, S9e, S9g, S9h, S9i, S9k, S9n, S9q, S9s, S9u and S9v) and only two biological properties were not retained (essential genes and mRNA degradation, Figures S9f and S9p) in the noisy networks. In summary, properties characterizing the KP-Net were retained to different levels in the noisy networks, showing that the characteristics of the KP-Net elucidated in this study represent the best of our knowledge to date about KP-Nets.

# Supplementary Figures

| 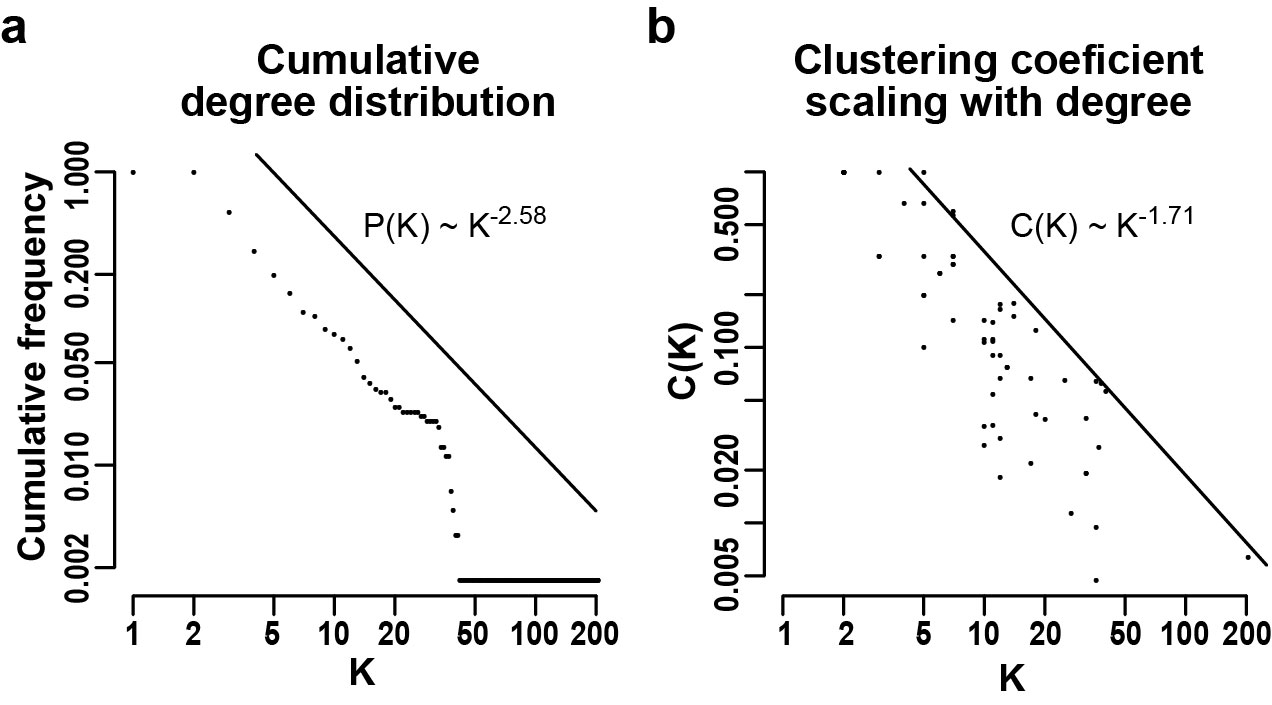 |
| --- |
| Figure S1. Distributions of the cumulative degree and of the clustering coefficient of KP-Net nodes follow a power law distribution. **(a)** The cumulative distribution of KP degrees (K) of the KP-Net plotted with logarithmically scaled axis showing that K follows a power law distribution: P(K) ~ K^-2.58^. **(b)** The scaling of KP clustering coefficients (C(K)) with the corresponding KP degrees (K) in the KP-Net plotted with a logarithmically scaled axis also showing that C(K) follows a power law distribution C(K) ~ K^-1.71^. |

| 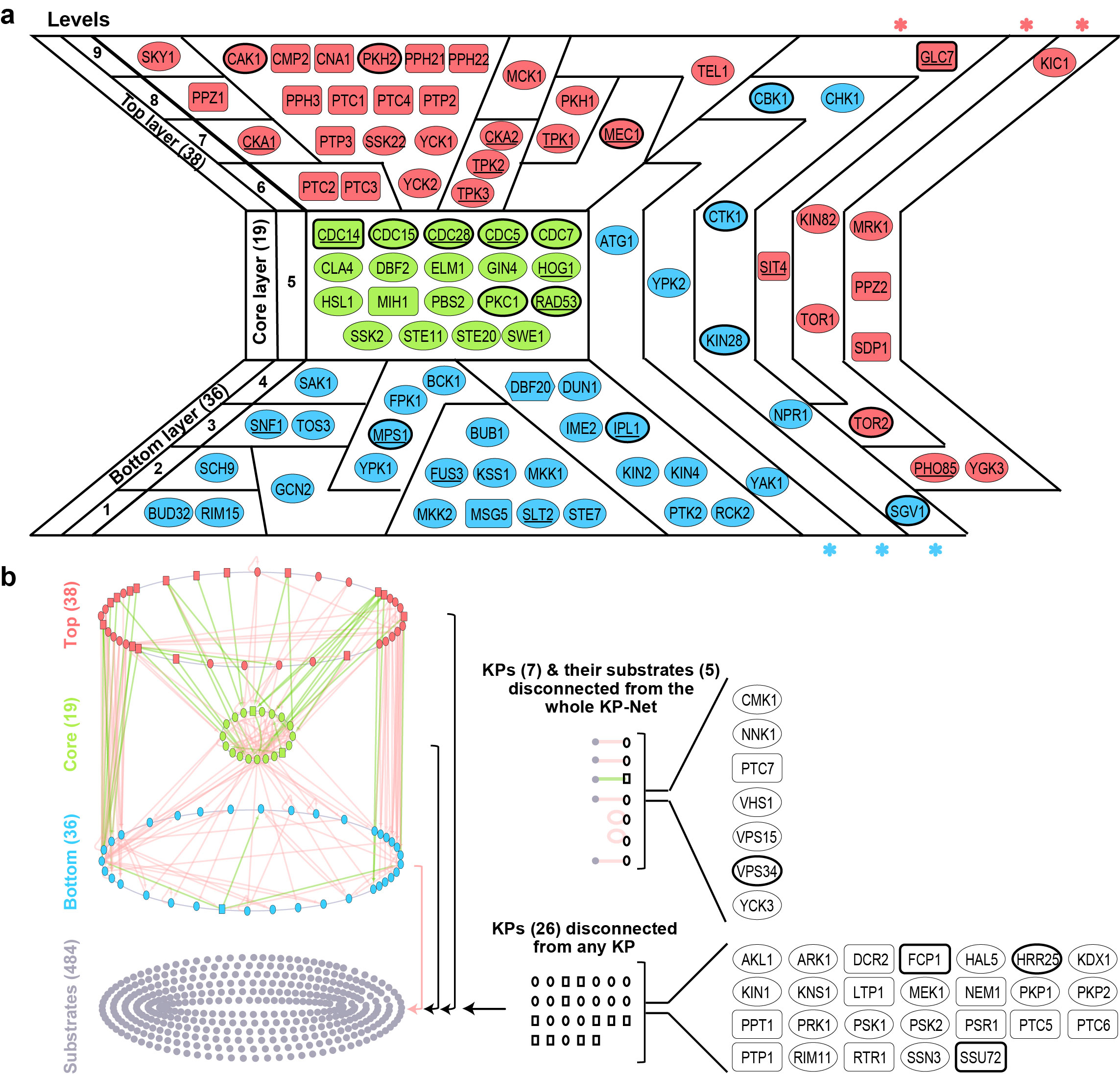 |
| --- |
| Figure S2. (a) The detailed hierarchical structure of the KP-Net. The classification of each KP in each level or range of levels after applying the VS algorithm on the KP-Net. Kinases are represented by an ellipse and phosphatases by a rectangle. Eleven and 9 nodes spanning the three layers are classified in top and bottom layers, respectively, because they are not regulated by and do not regulate any KP, respectively (nodes in columns with red and blue stars, respectively). Underlined KPs represent hubs and those with a thicker border designate essential KPs. (b) The relative position of the excluded KPs to the KP-Net hierarchy. The KP-Net hierarchy is shown at the left side of the figure and nodes that were excluded from this study analysis are shown with their identity at the right side of the figure. Seven KPs and their substrates (five proteins that are not KPs) were excluded from KP-Net analyses, because they are disconnected from the whole KP-Net. Twenty six KPs were also excluded from KP-Net analyses, because they do not regulate any KP and they are not regulated by any KP. Numbers between parentheses indicate the number of nodes and arrows represent directed interactions (red: phosphorylation, green: dephosphorylation and black: both). |

| 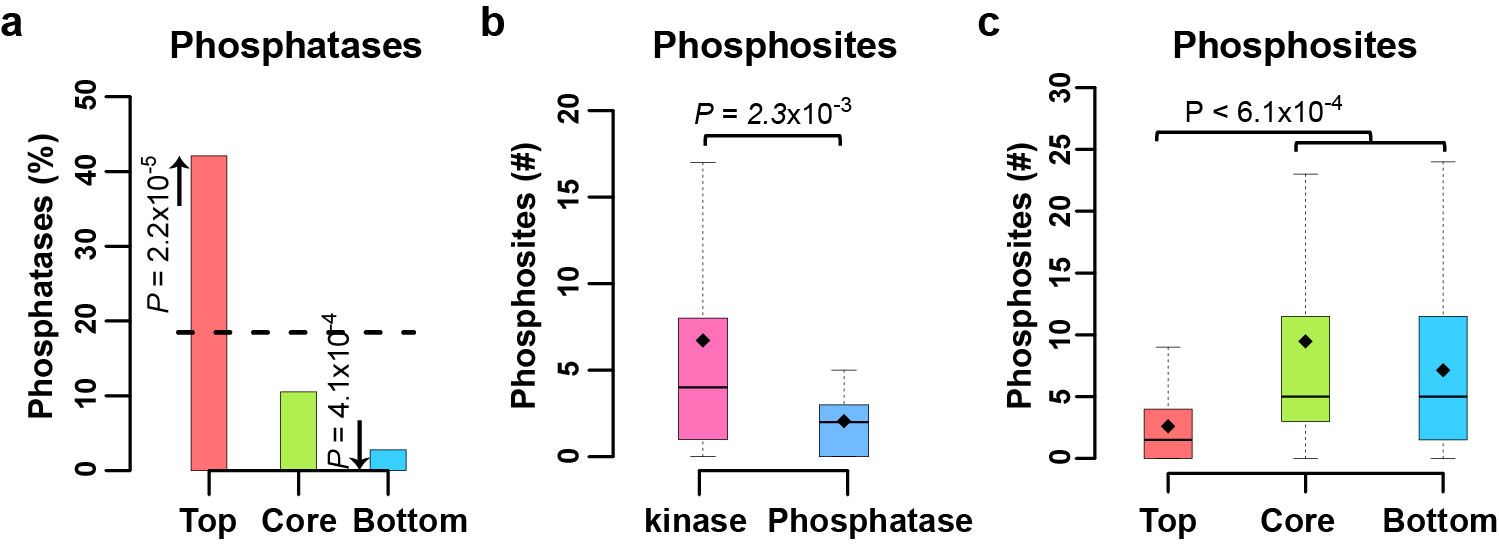 |
| --- |
| Figure S3. Percentage of phosphatases in each of the three layers of the KP-Net and distribution of phosphosites among KPs and among the different layers of the KP-Net. **(a)** Percentage of phosphatases in the different layers of the KP-Net. Distribution of phosphosites among **(b)** sequences of KPs belonging to the KP-Net and among **(c)** sequences of KPs in the different layers of the KP-Net. Phosphosites validated by at least two studies were taken from the PhosphoGRID database. The broken line in the bar plot represents the expected mean of phosphatases percentage in each layer. Black diamonds in box plots designate the average of the number of phosphosites in KPs and in each layer, respectively. Outliers were omitted from boxplots to simplify data representation. *P*-values were calculated by comparing property means of two layers and the enrichment/depletion of a property within a layer using the randomization test (RT, Methods) and the hypergeometric test (HT), respectively. For description of the used datasets see Supplementary Materials. |

| 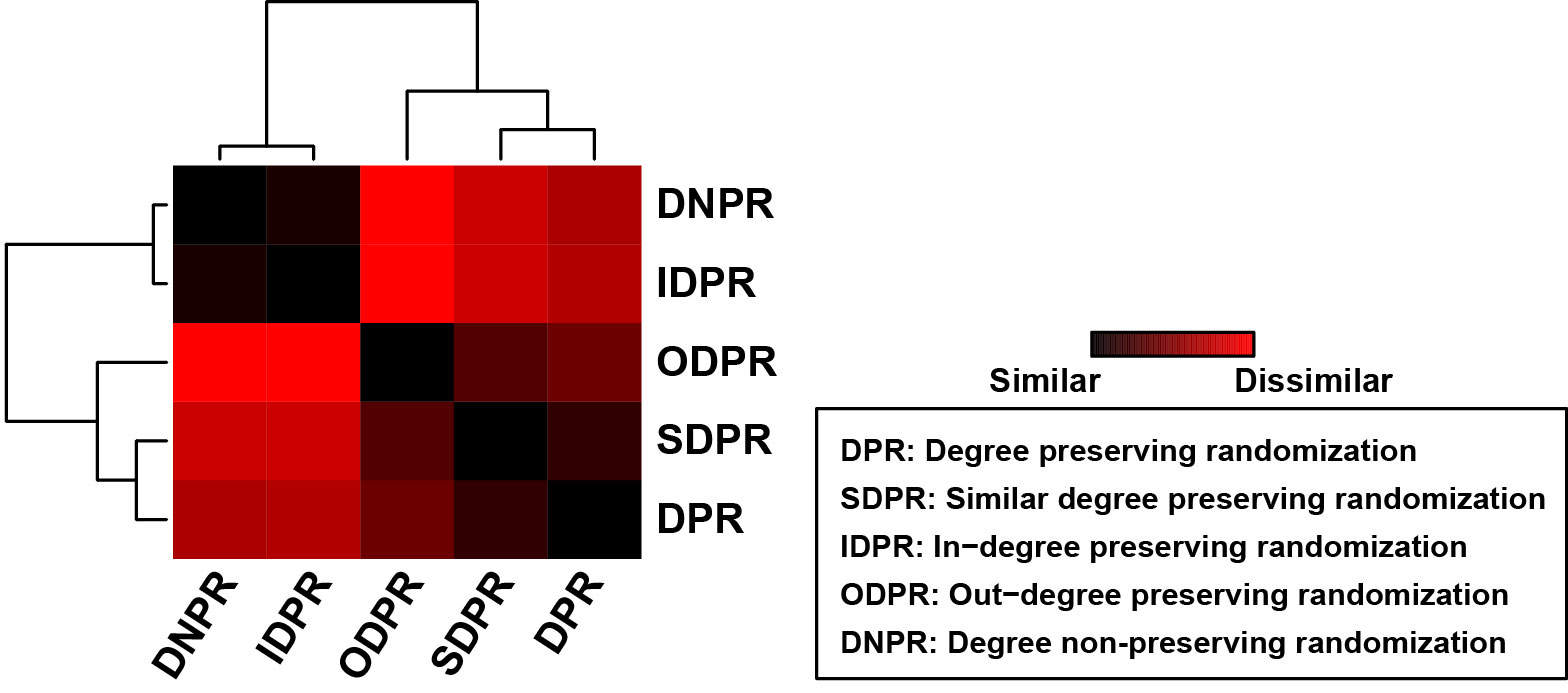 |
| --- |
| Figure S4. Clustering of the different sets of randomized networks. One thousand random networks were generated using each of the following randomization methods: degree preserving randomization (DPR, Methods), similar degree preserving randomization (SDPR, Methods), in-degree preserving randomization (IDPR, Methods), out-degree preserving randomization (ODPR, Methods) and degree non-preserving randomization (DNPR, Methods). Random networks were sorted using the VS algorithm. The Euclidean distance was then used to calculate dissimilarities between the different sets of random networks in terms of the studied properties of KPs in each layer of these random networks. Agglomerative clustering was performed using the average linkage criteria. |
|  |

| 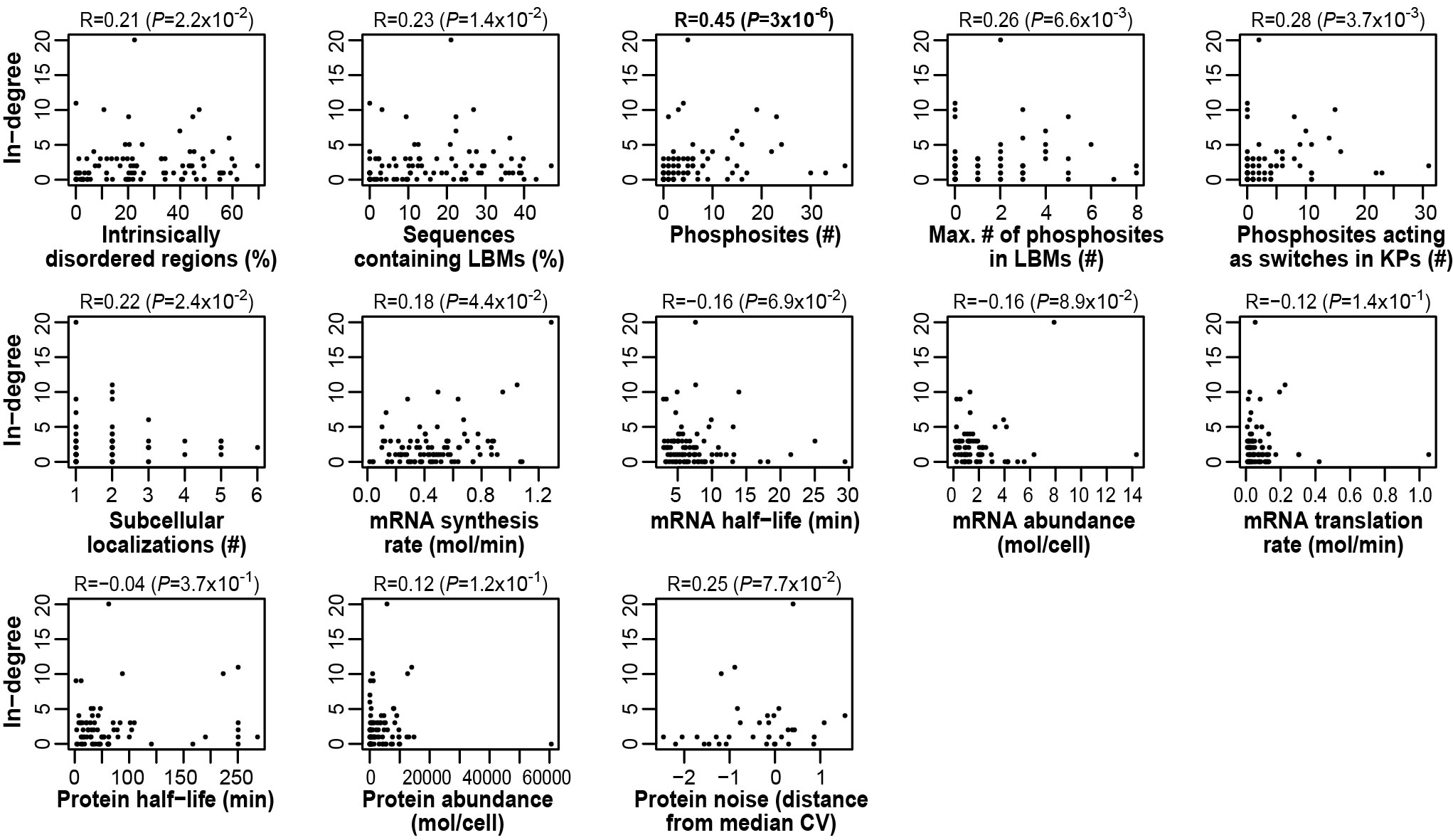 |
| --- |
| Figure S5. Correlation between KP in-degrees and KP numerical properties. Correlations were calculated using the Spearman correlation coefficient and *P*-values of these correlations are reported between parentheses. Values in bold indicate the presence of moderate correlations (between 0.3 and 0.6) with a *P-*value < 0.05. |

| 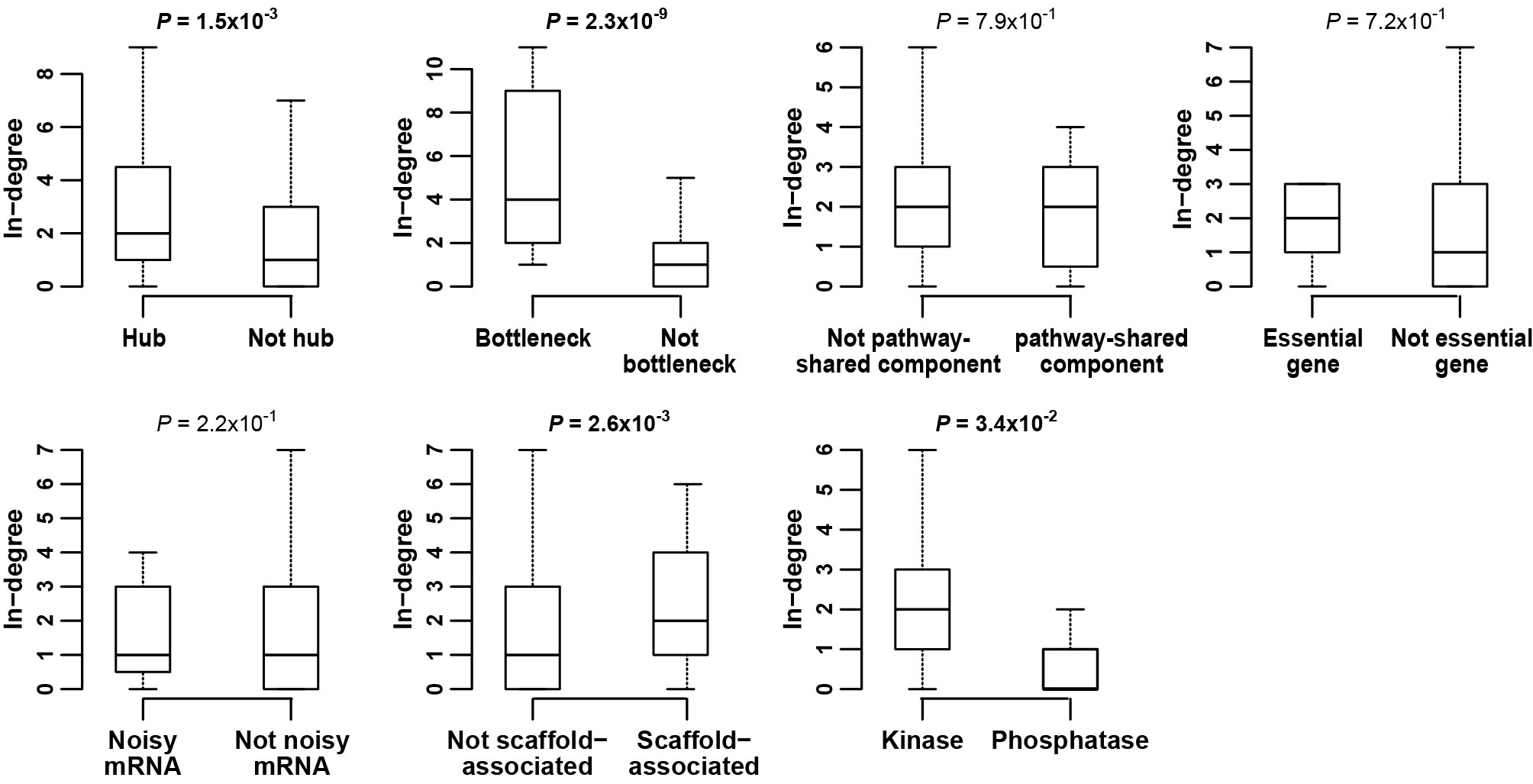 |
| --- |
| Figure S6. In-degree distribution of KPs characterized and not characterized by each of the studied categorical properties. The studied categorical properties are: hub, bottleneck, pathway-shared component, essential gene, noisy mRNA, scaffold-associated KP and type of enzyme (KP). *P*-value of the significance of the difference between in-degree means of KPs characterized by and not characterized by each of these properties were calculated using the one-way ANOVA test. Values in bold indicate significant *P-*values (< 0.05). |

| 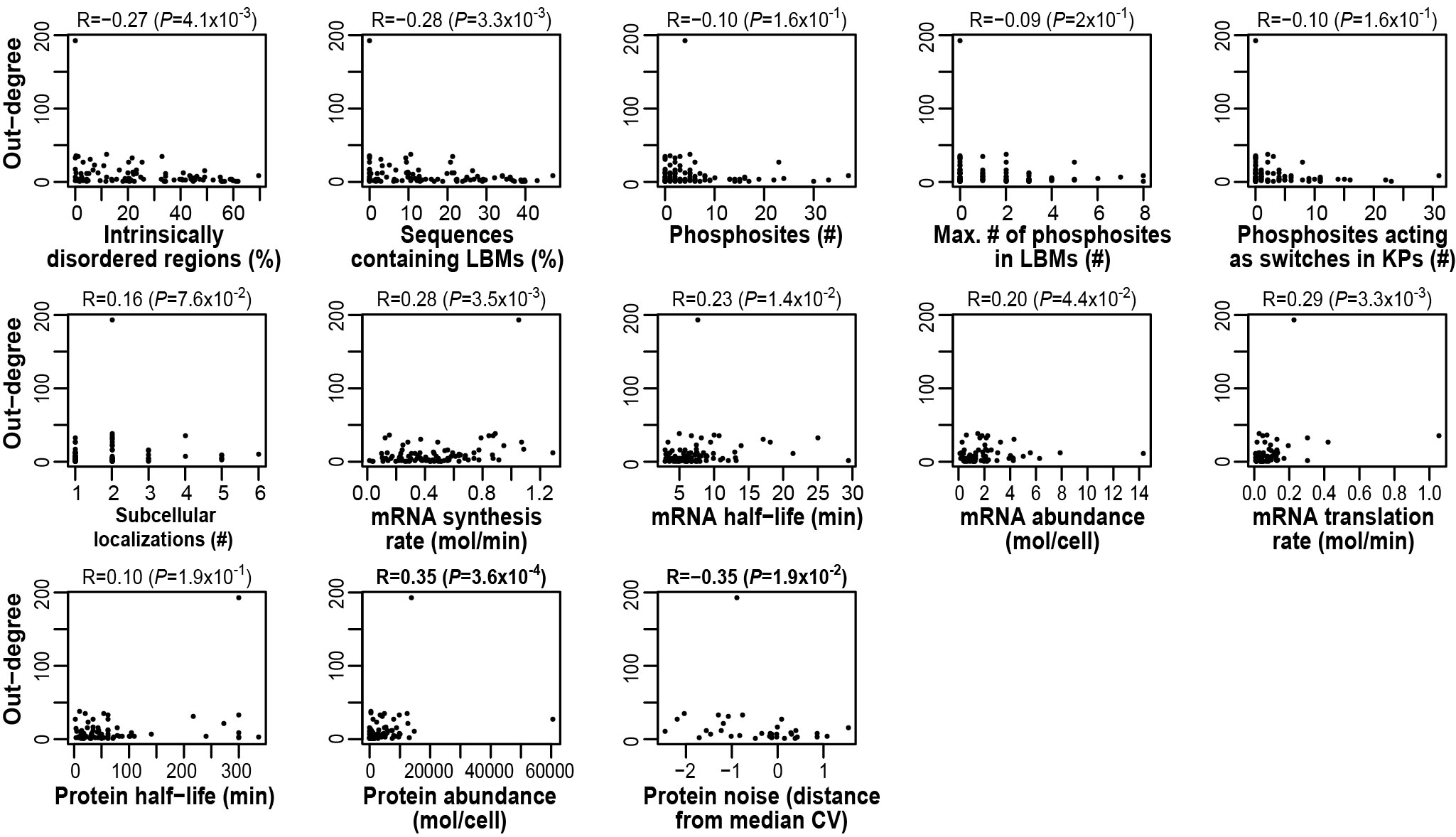 |
| --- |
| Figure S7. Correlation between KP out-degrees and KP numerical properties. Correlations were calculated using the Spearman correlation coefficient and *P*-values of these correlations are reported between parentheses. Values in bold indicate the presence of moderate correlations (between 0.3 and 0.6) with a *P-*value < 0.05. |

| 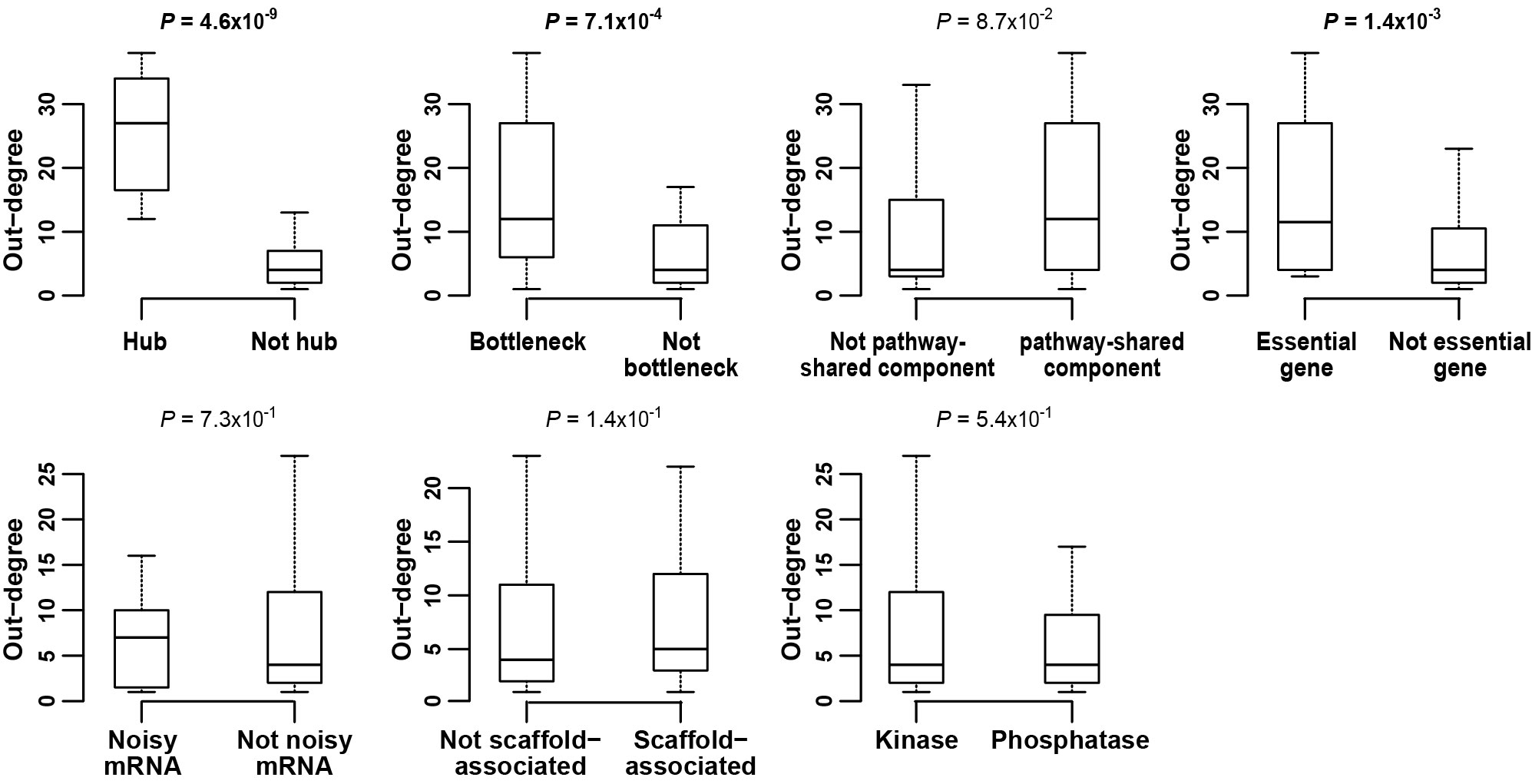 |
| --- |
| Figure S8. Out-degree distribution of KPs characterized and not characterized by each of the studied categorical properties. The studied categorical properties are: hub, bottleneck, pathway-shared component, essential gene, noisy mRNA, scaffold-associated and type of enzyme (KP). *P*-value of the significance of the difference between out-degree means of KPs characterized by and not characterized by each of these properties were calculated using the one-way ANOVA test. Values in bold indicate significant *P-*values (< 0.05). |


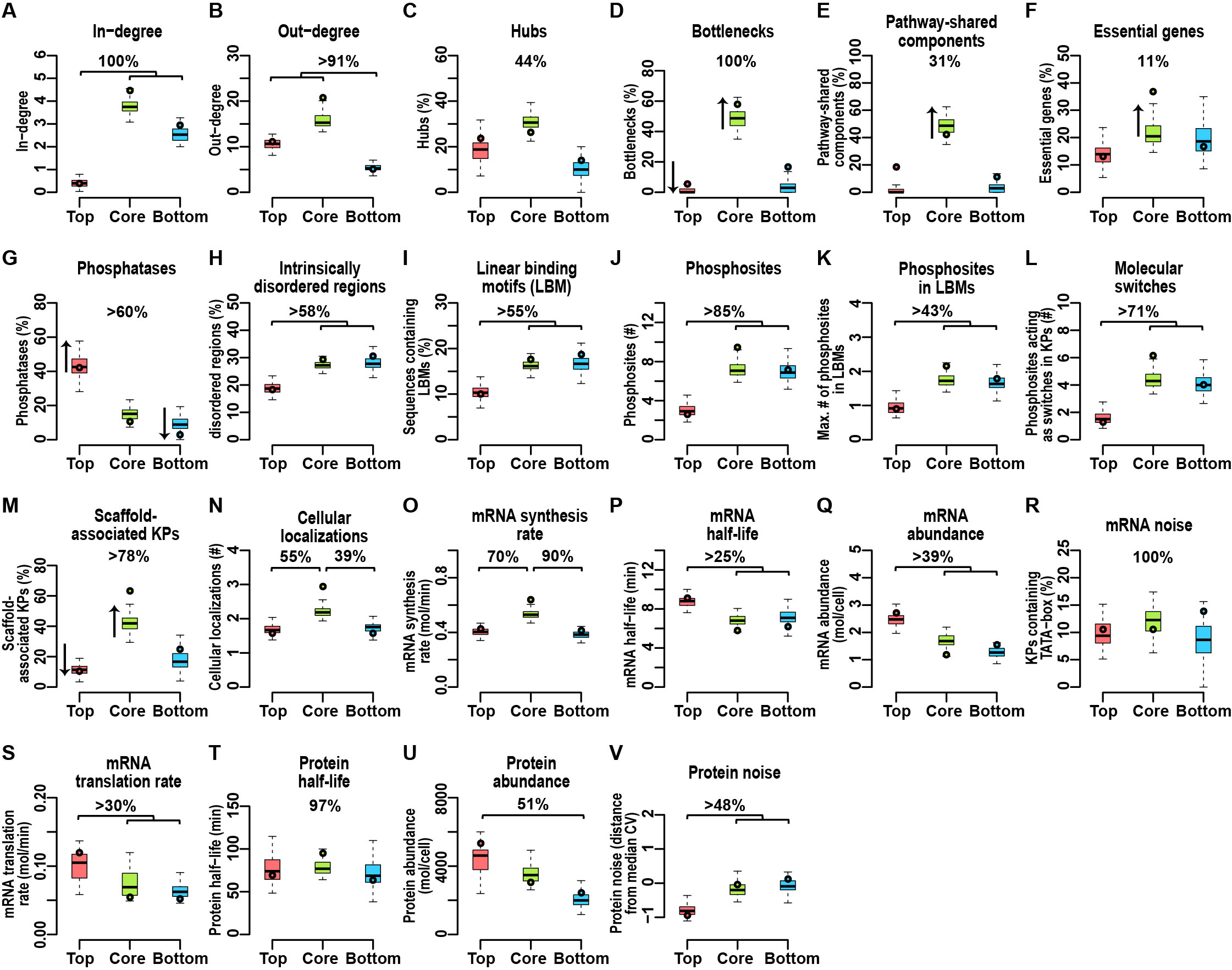


Figure S9. Distribution of the different properties of KPs in each layer of 100 noisy networks. Box plots represent the distribution of the means of KP properties in each layer of the 100 noisy networks that were generated from the KP-Net by randomly adding 200 edges. Coloured circles represent the mean of KP properties of KP-Net layers. Percentages at the top of each box plot represent the fraction of noisy networks that have significant (e.g. out-degree) and non-significant (e.g. hubs) differences among their layers. In other words percentages represent the fraction of noisy networks that their layer properties have the same pattern as layer properties of the KP-Net assembled in this study.

# Supplementary Tables

Table S1. Annotation of dephosphorylation interactions. The number of dephosphorylation interactions that were annotated in different public databases and in this study.

| **Database** | **Dephosphorylation interactions (#) as to March-2012** | **Dephosphorylation interactions (#) as to June-2016** |
| --- | --- | --- |
| Biogrid [[17](#_ENREF_17)] | 54 | 86 |
| IntAct [[29](#_ENREF_29)] | 6 | 8 |
| BIND*/BOND [[30](#_ENREF_30)] | 6 | -^*^ |
| MINT [[31](#_ENREF_31)] | 2 | 2 |
| MIPS [[32](#_ENREF_32)] | 1 | 1 |
| DIP [[33](#_ENREF_33)] | 0 | 0 |
| **This study** | **0** | **169** |

^*^ The BIND database was combined with the BOND database. Data is not accessible (technical problems with the BOND database server).

Table S2. K-Nets and KP-Nets studies. The list of high throughput studies that were interested in K-Nets and KP-Nets, in characteristics of these networks and in the biological properties that were investigated in these studies.

| **Study** | **Network type** | **Network characteristics** | | | |  | **Investigated biological properties** | | | | | | | | | | | | | | | | | | | | | | |
| --- | --- | --- | --- | --- | --- | --- | --- | --- | --- | --- | --- | --- | --- | --- | --- | --- | --- | --- | --- | --- | --- | --- | --- | --- | --- | --- | --- | --- | --- |
|  |  | **Causality** | **Directionality** | ***In vivo* interactions** | **Includes phosphatases** |  | **GO terms** | **Phenotype** | **Phosphorylation consensus motifs** | **Logic motifs** | **Disordered regions** | **Phosphosites** | **Evolution rank** | **Pathways** | **Essentiality** | **Conservation** | **Physical interaction** | **Genetic interaction** | **mRNA abundance** | **Protein abundance** | **mRNA degradation** | **Protein degradation** | **Protein noise** | **Linear binding motifs** | **Molecular switches** | **Interaction with scaffolds** | **mRNA synthesis rate** | **mRNA translation rate** | **mRNA noise** |
| Ptacek et al. 2005 (2) | Phosphorylation network | 1 | 1 | 0 | 0 |  | 1 | 0 | 1 | 1 | 0 | 1 | 0 | 0 | 0 | 0 | 0 | 0 | 0 | 0 | 0 | 0 | 0 | 0 | 0 | 0 | 0 | 0 | 0 |
| Fiedler et al. 2009 (3) | Genetic interaction network | 0 | 0 | 1 | 1 |  | 0 | 0 | 0 | 1 | 0 | 0 | 0 | 1 | 0 | 0 | 0 | 0 | 0 | 0 | 0 | 0 | 0 | 0 | 0 | 0 | 0 | 0 | 0 |
| Bodenmiller et al.2010 (4) | Genetic deletion network | 0 | 0 | 1 | 1 |  | 1 | 1 | 0 | 0 | 0 | 0 | 0 | 0 | 0 | 0 | 0 | 0 | 0 | 0 | 0 | 0 | 0 | 0 | 0 | 0 | 0 | 0 | 0 |
| Breitkreutz et al. 2010 (5) | Protein-protein interaction network | 0 | 0 | 1 | 1 |  | 1 | 0 | 0 | 0 | 1 | 1 | 0 | 0 | 0 | 0 | 0 | 0 | 0 | 0 | 0 | 0 | 0 | 0 | 0 | 0 | 0 | 0 | 0 |
| Bhardwaj et al. 2010 (6) | Co-Phosphorylation network | 1 | 1 | 0 | 0 |  | 1 | 0 | 0 | 1 | 0 | 0 | 1 | 0 | 0 | 0 | 0 | 0 | 0 | 0 | 0 | 0 | 0 | 0 | 0 | 0 | 0 | 0 | 0 |
| Sharifpoor et al. 2011 (7) | Kinase interaction network | 0 | 0 | 0/1 | 0 |  | 1 | 0 | 0 | 0 | 0 | 0 | 0 | 0 | 0 | 0 | 1 | 1 | 0 | 0 | 0 | 0 | 0 | 0 | 0 | 0 | 0 | 0 | 0 |
| Cheng et al. 2015 (1) | Phosphorylation network | 1 | 1 | 0 | 0 |  | 1 | 0 | 0 | 1 | 0 | 0 | 0 | 0 | 1 | 1 | 1 | 1 | 1 | 1 | 1 | 1 | 1 | 0 | 0 | 0 | 0 | 0 | 0 |
| This study | Phosphorylation network | 1 | 1 | 1 | 1 |  | 1 | 0 | 1 | 1 | 1 | 1 | 0 | 1 | 1 | 0 | 0 | 0 | 1 | 1 | 1 | 1 | 1 | 1 | 1 | 1 | 1 | 1 | 1 |

Table S3. Core layer KPs implicated in decision-making. KPs in the core layer that are implicated in decision-making processes and the list of these processes.

| **K/P gene name** | **Biological role** |
| --- | --- |
| Cdc14 | - Promotes the mitotic exit by counteracting mitotic Cdc28 activity [[34](#_ENREF_34)]. |
|  | - Regulates various microtubule-related processes at the metaphase/anaphase transition [[35](#_ENREF_35)]. |
| Cdc28 | - Drives the cell cycle in the budding yeast [[36](#_ENREF_36)]. |
|  | - Prevents catastrophic M-phase progression after DNA replication arrest [[37](#_ENREF_37)]. |
|  | - Arrests the spindle assembly checkpoint on spindle damage [[38](#_ENREF_38)]. |
| Cdc5 | Attenuates the DNA damage checkpoint enabling cells to adapt to this damage [[39](#_ENREF_39)]. |
| Cdc7 | Promotes the replication checkpoint [[40](#_ENREF_40)]. |
| Elm1 | Regulates spindle position checkpoint [[41](#_ENREF_41)]. |
| Gin4 and Hsl1 | Link entry into mitosis to proper septin organization [[42](#_ENREF_42)]. |
| Hog1 | Coordinates replication and transcription under osmotic stress [[43](#_ENREF_43)]. |
| Mih1 | Promotes anaphase onset [[44](#_ENREF_44)]. |
| Rad53 | Is involved in the three DNA damage checkpoints (i.e. G1/S transition, during the S phase and before mitosis) [[45](#_ENREF_45)]. |
| Swe1 | Enables the morphogenesis checkpoint to delay nuclear division in cells that have not budded [[46](#_ENREF_46)]. |

# References

1. SGD Project. <http://downloads.yeastgenome.org/curation/literature/archive/>. Aug. 2015.

2. Tenenbaum D. KEGGREST: Client-side REST access to KEGG. R package version 1.8.0.

3. R Core Team. R: A Language and Environment for Statistical Computing. 2012.

4. Huber W, Carey VJ, Gentleman R, Anders S, Carlson M, Carvalho BS et al. Orchestrating high-throughput genomic analysis with Bioconductor. Nat Methods. 2015;12(2):115-21. doi:10.1038/nmeth.3252.

5. Eser P, Demel C, Maier KC, Schwalb B, Pirkl N, Martin DE et al. Periodic mRNA synthesis and degradation co-operate during cell cycle gene expression. Molecular systems biology. 2014;10:717. doi:10.1002/msb.134886.

6. Sun M, Schwalb B, Schulz D, Pirkl N, Etzold S, Lariviere L et al. Comparative dynamic transcriptome analysis (cDTA) reveals mutual feedback between mRNA synthesis and degradation. Genome Res. 2012;22(7):1350-9. doi:10.1101/gr.130161.111.

7. Miura F, Kawaguchi N, Yoshida M, Uematsu C, Kito K, Sakaki Y et al. Absolute quantification of the budding yeast transcriptome by means of competitive PCR between genomic and complementary DNAs. BMC Genomics. 2008;9:574. doi:10.1186/1471-2164-9-574.

8. Wang M, Weiss M, Simonovic M, Haertinger G, Schrimpf SP, Hengartner MO et al. PaxDb, a database of protein abundance averages across all three domains of life. Molecular & cellular proteomics : MCP. 2012;11(8):492-500. doi:10.1074/mcp.O111.014704.

9. Arava Y, Wang Y, Storey JD, Liu CL, Brown PO, Herschlag D. Genome-wide analysis of mRNA translation profiles in Saccharomyces cerevisiae. Proceedings of the National Academy of Sciences of the United States of America. 2003;100(7):3889-94. doi:10.1073/pnas.0635171100.

10. Belle A, Tanay A, Bitincka L, Shamir R, O'Shea EK. Quantification of protein half-lives in the budding yeast proteome. Proceedings of the National Academy of Sciences of the United States of America. 2006;103(35):13004-9. doi:10.1073/pnas.0605420103.

11. Basehoar AD, Zanton SJ, Pugh BF. Identification and distinct regulation of yeast TATA box-containing genes. Cell. 2004;116(5):699-709.

12. Newman JR, Ghaemmaghami S, Ihmels J, Breslow DK, Noble M, DeRisi JL et al. Single-cell proteomic analysis of S. cerevisiae reveals the architecture of biological noise. Nature. 2006;441(7095):840-6. doi:10.1038/nature04785.

13. Charif D, Lobry JR. SeqinR 1.0-2: a contributed package to the R project for statistical computing devoted to biological sequences retrieval and analysis. In: Vendruscolo UBaMPaHERaM, editor. Structural approaches to sequence evolution: Molecules, networks, populations. vol Biological and Medical Physics, Biomedical Engineering. New York: Springer Verlag; 2007. p. 207-32.

14. SGD Project. <http://downloads.yeastgenome.org/sequence/S288C_reference/orf_protein/archive/>. Nov. 2014.

15. Stark C, Su TC, Breitkreutz A, Lourenco P, Dahabieh M, Breitkreutz BJ et al. PhosphoGRID: a database of experimentally verified in vivo protein phosphorylation sites from the budding yeast Saccharomyces cerevisiae. Database (Oxford). 2010;2010:bap026. doi:10.1093/database/bap026.

16. Sharifpoor S, Nguyen Ba AN, Young JY, van Dyk D, Friesen H, Douglas AC et al. A quantitative literature-curated gold standard for kinase-substrate pairs. Genome Biol. 2011;12(4):R39. doi:10.1186/gb-2011-12-4-r39.

17. Stark C, Breitkreutz BJ, Reguly T, Boucher L, Breitkreutz A, Tyers M. BioGRID: a general repository for interaction datasets. Nucleic Acids Res. 2006;34(Database issue):D535-9. doi:10.1093/nar/gkj109.

18. Magrane M, Consortium U. UniProt Knowledgebase: a hub of integrated protein data. Database (Oxford). 2011;2011:bar009. doi:10.1093/database/bar009.

19. Ba AN, Moses AM. Evolution of characterized phosphorylation sites in budding yeast. Molecular biology and evolution. 2010;27(9):2027-37. doi:10.1093/molbev/msq090.

20. Fiedler D, Braberg H, Mehta M, Chechik G, Cagney G, Mukherjee P et al. Functional organization of the S. cerevisiae phosphorylation network. Cell. 2009;136(5):952-63. doi:10.1016/j.cell.2008.12.039.

21. Muller HM, Kenny EE, Sternberg PW. Textpresso: an ontology-based information retrieval and extraction system for biological literature. PLoS biology. 2004;2(11):e309. doi:10.1371/journal.pbio.0020309.

22. Institute EB. CiteXplore. <http://www.ebi.ac.uk/citexplore>. Accessed 21-December-2013.

23. Clauset A, Shalizt C, Newman ME. Power-law distributions in empirical data. arXiv:07061062v2 [physicsdata-an]. 2009.

24. Lai W, Aldous D. Fitting Power Law Distributions to Data2012.

25. Falcon S, Gentleman R. Using GOstats to test gene lists for GO term association. Bioinformatics. 2007;23(2):257-8. doi:10.1093/bioinformatics/btl567.

26. Dosztanyi Z, Csizmok V, Tompa P, Simon I. The pairwise energy content estimated from amino acid composition discriminates between folded and intrinsically unstructured proteins. Journal of molecular biology. 2005;347(4):827-39. doi:10.1016/j.jmb.2005.01.071.

27. Meszaros B, Simon I, Dosztanyi Z. Prediction of protein binding regions in disordered proteins. PLoS Comput Biol. 2009;5(5):e1000376. doi:10.1371/journal.pcbi.1000376.

28. Kanshin E, Bergeron-Sandoval LP, Isik SS, Thibault P, Michnick SW. A cell-signaling network temporally resolves specific versus promiscuous phosphorylation. Cell reports. 2015;10(7):1202-14. doi:10.1016/j.celrep.2015.01.052.

29. Kerrien S, Aranda B, Breuza L, Bridge A, Broackes-Carter F, Chen C et al. The IntAct molecular interaction database in 2012. Nucleic Acids Res. 2012;40(Database issue):D841-6. doi:10.1093/nar/gkr1088.

30. Bader GD, Betel D, Hogue CW. BIND: the Biomolecular Interaction Network Database. Nucleic Acids Res. 2003;31(1):248-50.

31. Licata L, Briganti L, Peluso D, Perfetto L, Iannuccelli M, Galeota E et al. MINT, the molecular interaction database: 2012 update. Nucleic Acids Res. 2012;40(Database issue):D857-61. doi:10.1093/nar/gkr930.

32. Mewes HW, Frishman D, Guldener U, Mannhaupt G, Mayer K, Mokrejs M et al. MIPS: a database for genomes and protein sequences. Nucleic Acids Res. 2002;30(1):31-4.

33. Salwinski L, Miller CS, Smith AJ, Pettit FK, Bowie JU, Eisenberg D. The Database of Interacting Proteins: 2004 update. Nucleic Acids Res. 2004;32(Database issue):D449-51. doi:10.1093/nar/gkh086.

34. Visintin R, Craig K, Hwang ES, Prinz S, Tyers M, Amon A. The phosphatase Cdc14 triggers mitotic exit by reversal of Cdk-dependent phosphorylation. Molecular cell. 1998;2(6):709-18.

35. Chiroli E, Rancati G, Catusi I, Lucchini G, Piatti S. Cdc14 inhibition by the spindle assembly checkpoint prevents unscheduled centrosome separation in budding yeast. Molecular biology of the cell. 2009;20(10):2626-37. doi:10.1091/mbc.E08-11-1150.

36. Enserink JM, Kolodner RD. An overview of Cdk1-controlled targets and processes. Cell division. 2010;5:11. doi:10.1186/1747-1028-5-11.

37. Enserink JM, Hombauer H, Huang ME, Kolodner RD. Cdc28/Cdk1 positively and negatively affects genome stability in S. cerevisiae. The Journal of cell biology. 2009;185(3):423-37. doi:10.1083/jcb.200811083.

38. Kitazono AA, Garza DA, Kron SJ. Mutations in the yeast cyclin-dependent kinase Cdc28 reveal a role in the spindle assembly checkpoint. Molecular genetics and genomics : MGG. 2003;269(5):672-84. doi:10.1007/s00438-003-0870-y.

39. Vidanes GM, Sweeney FD, Galicia S, Cheung S, Doyle JP, Durocher D et al. CDC5 inhibits the hyperphosphorylation of the checkpoint kinase Rad53, leading to checkpoint adaptation. PLoS biology. 2010;8(1):e1000286. doi:10.1371/journal.pbio.1000286.

40. Ogi H, Wang CZ, Nakai W, Kawasaki Y, Masumoto H. The role of the Saccharomyces cerevisiae Cdc7-Dbf4 complex in the replication checkpoint. Gene. 2008;414(1-2):32-40. doi:10.1016/j.gene.2008.02.010.

41. Caydasi AK, Kurtulmus B, Orrico MI, Hofmann A, Ibrahim B, Pereira G. Elm1 kinase activates the spindle position checkpoint kinase Kin4. The Journal of cell biology. 2010;190(6):975-89. doi:10.1083/jcb.201006151.

42. Barral Y, Parra M, Bidlingmaier S, Snyder M. Nim1-related kinases coordinate cell cycle progression with the organization of the peripheral cytoskeleton in yeast. Genes & development. 1999;13(2):176-87.

43. Duch A, Felipe-Abrio I, Barroso S, Yaakov G, Garcia-Rubio M, Aguilera A et al. Coordinated control of replication and transcription by a SAPK protects genomic integrity. Nature. 2013;493(7430):116-9. doi:10.1038/nature11675.

44. Lianga N, Williams EC, Kennedy EK, Dore C, Pilon S, Girard SL et al. A Wee1 checkpoint inhibits anaphase onset. The Journal of cell biology. 2013;201(6):843-62. doi:10.1083/jcb.201212038.

45. Foiani M, Pellicioli A, Lopes M, Lucca C, Ferrari M, Liberi G et al. DNA damage checkpoints and DNA replication controls in Saccharomyces cerevisiae. Mutation research. 2000;451(1-2):187-96.

46. King K, Kang H, Jin M, Lew DJ. Feedback control of Swe1p degradation in the yeast morphogenesis checkpoint. Molecular biology of the cell. 2013;24(7):914-22. doi:10.1091/mbc.E12-11-0812.
